# Metastatic recurrence in women diagnosed with non-metastatic breast cancer: a systematic review and meta-analysis

**DOI:** 10.1186/s13058-024-01881-y

**Published:** 2024-11-27

**Authors:** Eileen Morgan, Colette O’Neill, Richa Shah, Oliver Langselius, Yaqi Su, Clara Frick, Hanna Fink, Aude Bardot, Paul M. Walsh, Ryan R. Woods, Lou Gonsalves, Jan F. Nygård, Serban Negoita, Esmeralda Ramirez-Pena, Karen Gelmon, Nicoleta Antone, Miriam Mutebi, Sabine Siesling, Fatima Cardoso, Julie Gralow, Isabelle Soerjomataram, Melina Arnold

**Affiliations:** 1 Cancer Surveillance Branch, International Agency for Research on Cancer (IARC/WHO), Lyon, France; 2 National Cancer Registry Ireland, Cork, Ireland; 3 BC Cancer, Vancouver, BC Canada; 4 Connecticut Department of Public Health, Connecticut Tumor Registry, Hartfort, CT USA; 5 Cancer Registry of Norway, Norwegian Institute of Public Health, Oslo, Norway; 6 Data Quality, Analysis, and Interpretation Branch, Division of Cancer Control and Population Sciences, Surveillance Research Program, National Cancer Institute, National Institutes of Health, Bethesda, MD USA; 7 Breast Cancer Center, Institute of Oncology “Ion Chiricuta”, Cluj-Napoca-Napoca, Romania; 8 Breast Surgical Oncology, Aga Khan University Hospital, Nairobi, Kenya; 9 Department of Research and Development, Netherlands Comprehensive Cancer Organisation (IKNL), Utrecht, The Netherlands; 10 Department of Health Technology and Services Research, Technical Medical Centre, University of Twente, Enschede, The Netherlands; 11 Breast Unit, Champalimaud Clinical Center/Champalimaud Foundation and ABC Global Alliance, Lisbon, Portugal; 12 American Society of Clinical Oncology, Alexandria, VA USA

**Keywords:** Systematic review, Meta-analysis, Breast cancer, Recurrence, Metastasis

## Abstract

**Background:**

To assess proportions of metastatic recurrence in women initially diagnosed with non-metastatic breast cancer by stage at diagnosis, breast cancer subtype, calendar period and age.

**Methods:**

A systematic search of MEDLINE and Web of Science databases (January 2010–12 May 2022) was conducted. Studies reporting the proportion of distant metastatic recurrence in women with non-metastatic breast cancer were identified and outcomes and characteristics were extracted. Risk of bias was assessed independently by two reviewers. Random-effects meta-analyses of proportions were used to calculate pooled estimates and 95% confidence intervals (CIs).

**Results:**

193 studies covering over 280,000 patients were included in the main analysis. Pooled proportions of metastatic recurrence increased with longer median follow-up time from 12.2% (95% CI 10.5–14.0%) at 1–4 years post diagnosis, 14.3% (95% CI 12.9–15.7%) at 5–9 years to 23.3% (95% CI 20.1–26.8) at 10 years or more. Regional variation was observed with pooled estimates ranging from 11.0% (95% CI 8.5–13.7%) in Europe to 26.4% (95% CI 16.7–37.4%) in Africa (1–4 years follow-up). Proportions of recurrence were higher in studies with diagnosis before 2000 (22.2%, 95% CI 15.1–30.3) compared to studies with diagnosis from 2000 onwards (12.8%, 95% CI 11.7–14.0). At 1–4 years median follow-up, pooled proportions of metastatic recurrence were higher in women with hormone receptor negative (15.2%, 95% CI 12.0–18.7%) compared with receptor positive disease (9.6%, 95% CI 6.2–13.6%) and in women with locally advanced (33.2%, 95% CI 24.7–42.3%) relative to early disease at initial diagnosis (4.8%, 95% CI 2.5–7.8%). Proportions were higher in those under 50 years compared with 70+ years, 18.6% (95% CI 15.9–21.4%) versus 13.3% (95% CI 9.2, 18.0%), respectively. Heterogeneity was high in all meta-analyses and results should be interpreted with caution.

**Conclusions:**

Higher proportions of metastatic recurrence in patients initially diagnosed at an advanced stage and in earlier calendar period emphasises the importance of early detection and treatment advancements. As the global number of breast cancer survivors increases, research and health policy efforts should be directed towards timely diagnosis and access to effective treatments and care.

*Study registration*: PROSPERO CRD42022314500.

**Supplementary Information:**

The online version contains supplementary material available at 10.1186/s13058-024-01881-y.

## Background

Breast cancer is the most common cancer diagnosed worldwide in women with an estimated 2.3 million new cases, and almost 700,000 deaths in 2022 [1, 2]. As both incidence and survival continue to increase in many world regions, the number of women living with a history of breast cancer (i.e. prevalence) continues to rise steadily and currently amounts to an estimated 8.2 million globally [2]. This number comprises the survivors but also the patients living with metastatic disease. Almost all breast-cancer related deaths are due to distant metastatic spread of the tumour, which remains incurable albeit treatable. While, in high-income countries, only a minority of breast cancer patients present with distant metastases at diagnosis (so called de novo), most cases of metastatic breast cancer (MBC) occur as recurrent disease after an initial non-distant-metastatic breast cancer diagnosis, treatment with curable intent and a disease-free period. To-date, the prevalence of recurrent MBC remains largely unknown, as historically population-based cancer registries (PBCRs) do not routinely collect detailed long-term follow-up and recurrence data and no international guidelines or definitions have been established. Yet, it is important to quantify the burden of MBC to evaluate the effectiveness of treatment, assess and compare outcomes on the population level, and to improve the allocation of resources as well as respond to the need for more information for persons living with this disease. Although studies have investigated proportions of (distant) recurrence in breast cancer patients, these are often based on treatment-/site- specific groups, which are small in sample size and not representative of all breast cancer patients. Randomized controlled trials (RCTs) have played an important role in the understanding of MBC [3], however, trials are often limited with shorter follow-up time and not representative of the general population compared to population-based studies. Recently, the Lancet Breast Cancer Commission called for the global collection of high-quality cancer registry data on cancer relapses as one of the key points for change [4]. To date, no effort has been made to compile and assess the knowledge on the proportion of distant recurrence, or the timing of recurrence, in women initially diagnosed with non-metastatic breast cancer on a large scale.

In this study, we conducted a systematic review of the literature and meta-analyses of studies that have measured proportions of distant recurrence in women diagnosed with an initial non-metastatic breast cancer and assessed patterns of distant recurrence in daily clinical practice.

## Methods

### Search strategy

A search strategy was developed (eTable 1 in the Supplement) and a literature search carried out using Pubmed and Web of Science. Articles were restricted to those published between January 2010 and 12 May 2022.

### Study selection

The screening process was divided and conducted independently by two pairs of reviewers (EM, CON, MA and AB) using Covidence Online Software (https://www.covidence.org). Firstly, titles and abstracts were screened and those that were deemed irrelevant by both reviewers were immediately excluded. Where there was a screening conflict between two reviewers, these were discussed and consulted with a third and fourth reviewer and a final decision was made. Following this, full text papers were requested and assessed for inclusion or exclusion.

### Inclusion and exclusion criteria

The following eligibility criteria were applied: (i) the study included women of any age with invasive non-metastatic breast cancer (ICD10 C50) as initial diagnosis; (ii) full research articles presenting findings from cohort or case–control studies, excluding RCTs; (iii) the study assessed the proportion of distant metastatic recurrences [5]; (iv) there was sufficient statistical information on the outcome of interest reporting both number of distant metastatic recurrences and total number of breast cancer cases in the population under study; (v) articles were published in the English language. Studies that provided only combined estimates for all recurrences, grouping distant metastases, loco-regional recurrences and second primary breast cancers, were excluded, as were studies including patients with in-situ or metastatic cancer at baseline. Although previous studies have investigated recurrence in breast cancer patients, these have been based on clinical trials which are not representative of the general population [3]. As such, RCTs, microarray studies, or studies that investigated specific diagnostic procedures were not included. Studies were also excluded when data were only presented as survival curves without accompanying estimates. Two reviewers reviewed studies that were from the same hospital or PBCR to check for overlap and included the study with the more recent period of diagnosis and longer follow-up. If an overlapping study provided additional data by another sub-group, these were included.

### Data extraction

Data extraction was conducted using a predesigned template including study design, author name, year of publication, location of the study, study setting, number of patients included, mean age at diagnosis, diagnosis period, specific breast cancer subtype, molecular groups, stage at diagnosis, hormonal status, median follow-up time, number of metastatic recurrences. Extraction was conducted by two investigators (EM and CON).

### Risk of bias assessment

Risk of bias of the included studies was assessed independently by pairs of two investigators (YS, RS, OL, HF, CF) using an adaptation of a specific tool for prevalence studies [6]. Articles were confirmed as having low risk or high risk of bias based on nine questions assessing bias in sampling, information detection and reporting: 1. Was the study population a close representation of the target population in relation to relevant variables? 2. Was the sampling frame a true or close representation of the target population? 3. Was some form of random selection used to select the sample, or was a population-based study undertaken? 4. Was the likelihood of missing information on distant recurrence and completeness of follow-up minimal? 5. Were data collected directly from patient examination, record linkage or scrutinising medical records? 6. Was an acceptable definition of distant recurrence used in the study? 7. Was the study instrument that measured the parameter of interest shown to be reliable and valid? 8. Was the same mode of data collection used for all subjects? 9. Were the numerator(s) and denominator(s) for the parameter of interest appropriate? If a study scored high in one of the nine questions, it was assigned with an overall high risk of bias score. Where high or low risk of bias could not be determined, moderate risk of bias was assigned.

### Statistical analysis

A random-effects meta-analysis of proportions, with the Freeman–Tukey double arcsine transformation, was used to examine the proportions of metastatic recurrence in women with breast cancer. A χ^2^-test for heterogeneity was calculated and the I^2^ statistic was determined to estimate the proportion of variation between study results attributable to heterogeneity rather than chance [7].

Random effects meta-analyses were used to examine the proportion of metastatic recurrence according to study setting (PBCR; hospital; other), median follow-up time (1–4; 5–9; 10+ years), calendar period of diagnosis (pre- and post- 1999), stage at diagnosis (early, stage I-IIa; locally advanced, stage IIb-IIIc), breast cancer subtype (hormone receptor positive [HR+]; hormone receptor negative [HR−]), age at diagnosis (< 50; 50–69; 70+ years) and region (Europe and South America; North America and Oceania; Asia; Africa). HR+ tumours were defined as tumours with estrogen receptors (ER+) and/ or progesterone receptors (PR+), HR- tumours were defined as tumours that did not contain estrogen or progesterone receptors. Details of cut-offs used by each study to define receptor positivity were not extracted for this review. Sensitivity analyses were conducted including studies that were rated as having low risk of bias.

The analysis was conducted using the *metaprop* command using Stata 14 software (Stata Corporation, College Station, Texas, USA).

### PROSPERO registration

This systematic review was registered in PROSPERO with the registration number CRD42022314500 (https://www.crd.york.ac.uk/prospero/display_record.php?RecordID=314500).

### Patient and public involvement

No patients or members of the public were involved in the design, conduct, or reporting of the study, or in the dissemination of findings. During their clinical routine, however, several of the authors have regular contact with patients with breast cancer during which clinical factors such as breast cancer subtype, treatment and follow-up for recurrence of breast cancer are discussed. The experiences from these interactions have been taken into account during the planning, conduct, and reporting of this study.

## Results

### Characteristics of included studies

The database search using PubMed and Web of Science yielded 10,138 articles to be screened (eFigure 1 in the Supplement). After screening titles and abstracts, 8,668 articles were excluded, with 1470 full text articles remaining to be screened for eligibility with the following reasons for exclusion applied: (1) wrong outcomes (mainly when locoregional and distant recurrence data were inseparable); (2) wrong patient population; (3) wrong study design; (4) missing or incorrect data reported; (5) overlapping data; (6) full text was not available in English; (7) duplicate and (8) multiple reasons, leaving 217 eligible articles to be included.

Appendix, Table 2 shows the characteristics of the 217 eligible studies. They were predominantly from hospital-based institutions (n = 185), followed by population-based cancer registries (n = 23) and an additional 9 from other settings (e.g. claims databases). Almost all studies were cohort in design (n = 213) and the rest were case-control (n = 2) or case series (n = 2). Most studies were from Asia (n = 88), followed by Europe (n = 71), North America (n = 43), Africa (n = 6), Oceania (n = 5), and South America (n = 4). Median follow-up duration for the included studies ranged from 1.2 to 15 years with most having 1–4 years (n = 102) or 5–9 years (n = 101) median follow-up, and the remaining with 10+ years (n = 14). There were 175 of the 217 (81%) eligible studies that reported how recurrence data were assessed. Most of these, (n = 170, 97%) used medical records containing clinical and/ or pathological information to confirm recurrence. The remaining studies used a combination of medical records and contacting patient to obtain complete information (n = 4), one study was patient reported.

### Overall proportions of distant recurrence in women with non-metastatic breast cancer

**Fig. 1:**
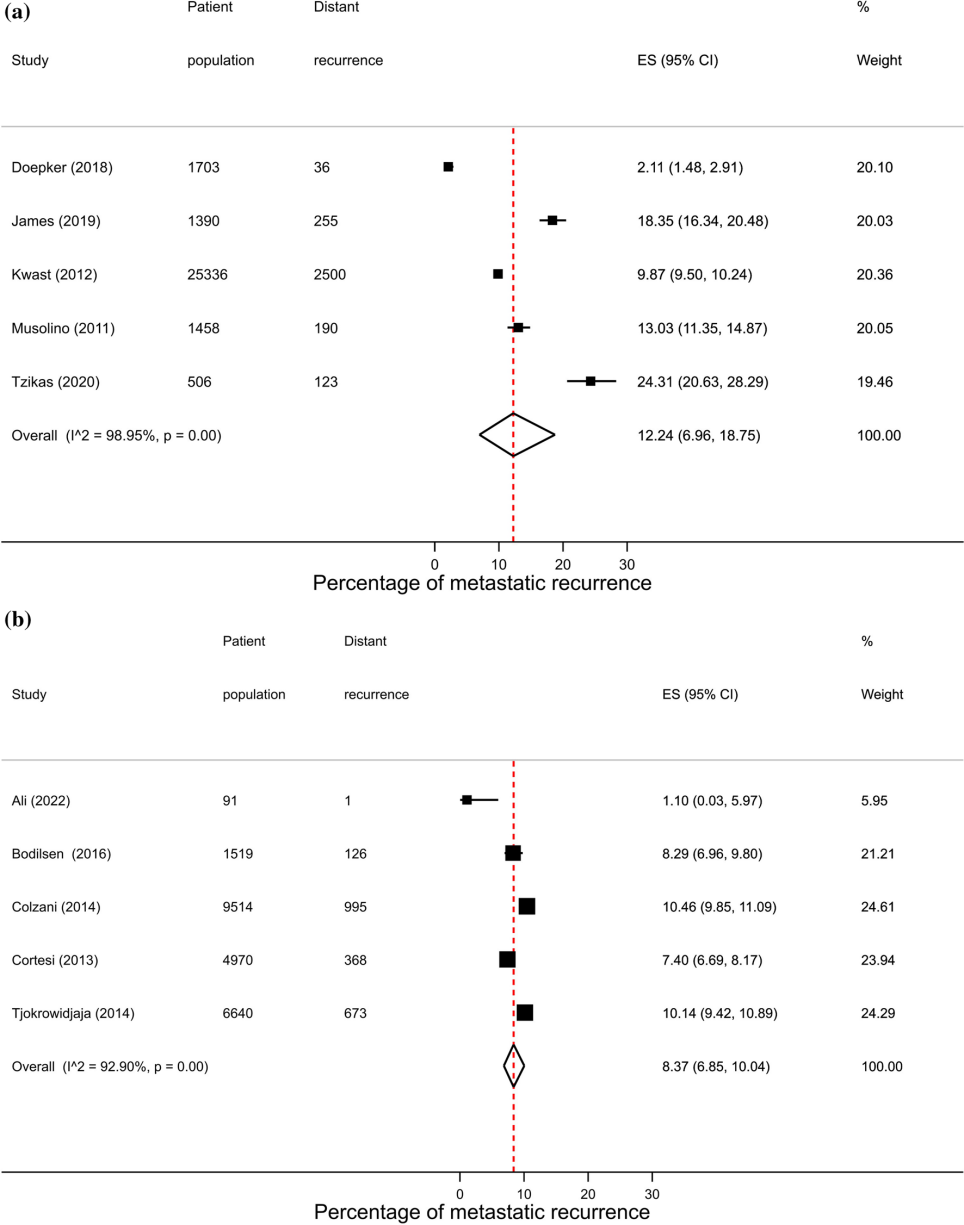

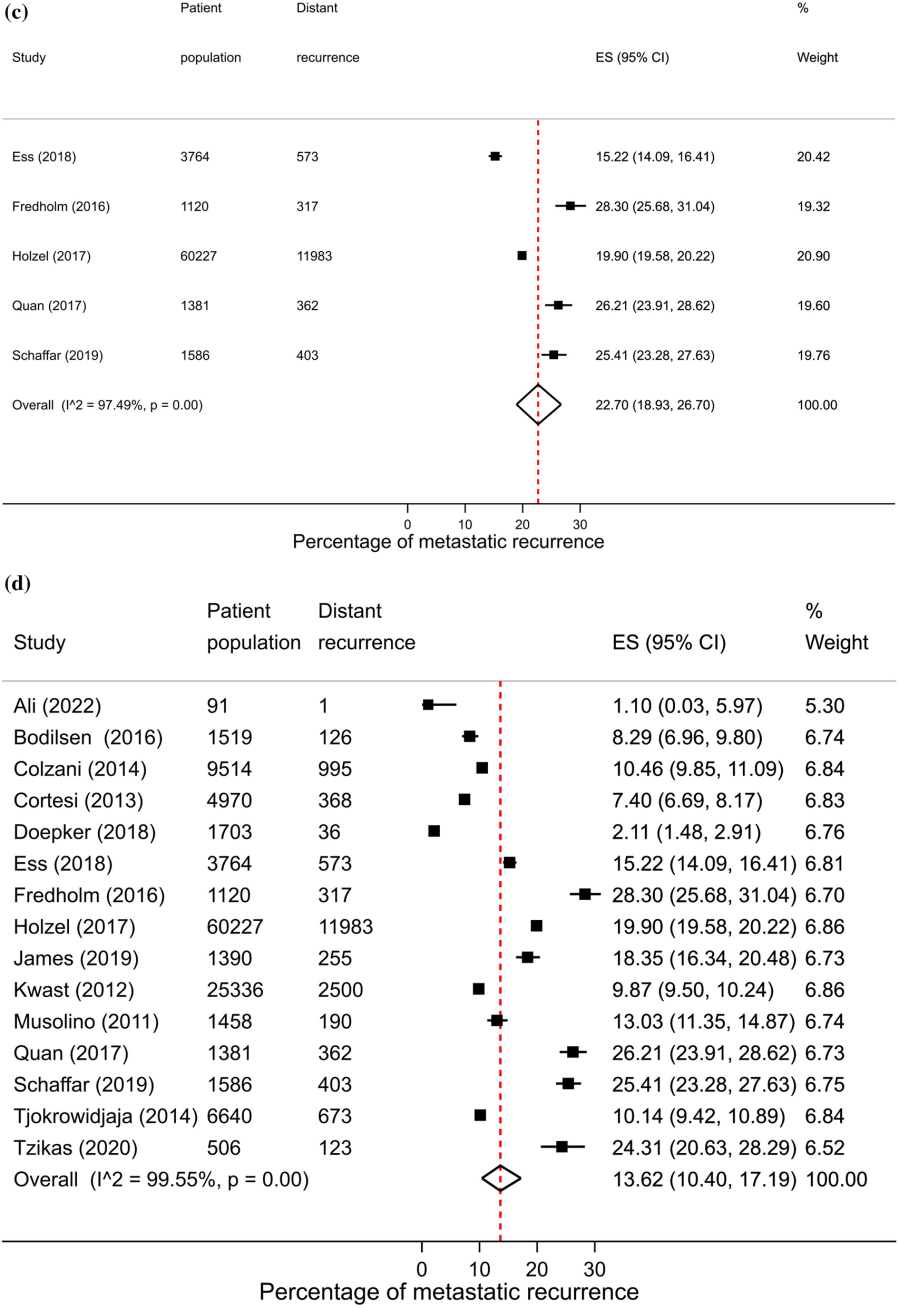
Forest plot of the proportion of metastatic recurrences in women diagnosed with breast cancer from PBCR studies at **a** 1–4 years; **b** 5–9 years; **c** 10+ years median follow-up and **d** overall

After removal of overlapping studies, 193 studies including 283,110 patients were included in the overall analysis. As similar proportions of recurrence were observed across hospital, PBCR (Fig. 1) and other settings (14.1% vs. 13.6% vs. 10.0%, respectively), results will focus on estimates combining these different source settings. The proportion of metastatic recurrence increased according to median follow-up time from 12.2% (95% CI 10.5–14.0%) at 1–4 years, 14.3% (95% CI 12.9–15.7%) at 5–9 years, and 23.3% (95% CI 20.1–26.8) at 10 years or more (Table 1). Due to marked differences in proportions of recurrence with increasing follow-up time, results will be presented by median follow-up time.

**Table 1:** Pooled proportions of metastatic recurrence rates in women diagnosed with breast cancer by median follow-up

	Median follow-up time							
	1–4 years		5–9 years		10 + years		Total	
	Studies (n)	Pooled proportion (95% CI)	Studies (n)	Pooled proportion (95% CI)	Studies (n)	Pooled proportion (95% CI)	Studies (n)^a^	Pooled proportion (95% CI)
Overall	95	12.2 (10.5–14.0)	88	14.3 (12.9–15.7)	13	23.3 (20.1–26.8)	190	13.9 (12.8–15.1)
Source setting								
Hospital	91	12.2 (10.3–14.2)	78	15.3 (13.7–16.9)	7	23.8 (18.2–29.8)	171	14.1 (12.8–15.5)
PBCR	5	12.2 (7–18.8)	5	8.4 (6.8–10)	5	22.7 (18.9–26.7)	15	13.6 (10.4–17.2)
Other	0	–	7	10.0 (5.7–15.4)	1	–	7	10.0 (5.7–15.4)
Region								
Africa	5	26.4 (16.7–37.4)	1	–	0	–	6	21.7 (11.7–33.8)
Asia	39	11.8 (9.2–14.7)	43	16.1 (13.9–18.4)	2	–	80	14.6 (12.6–16.7)
Europe	17	11.0 (8.5–13.7)	31	13.2 (11.4–15)	9	21.9 (19–24.9)	55	13.8 (12.1–15.7)
North America	29	10.2 (6.7–14.3)	10	7.7 (4.8–11.2)	2	–	41	10.2 (7.5–13.2)
Oceania	2	–	2	–	0	–	4	17.9 (10–27.5)
South America	3	22.6 (20.7–24.6)	1	–	0	–	4	31.5 (18.9–45.6)
Calendar year of diagnosis								
Until 1999	1	–	3	22.2 (15.6–29.6)	2	–	6	22.2 (15.1–30.3)
From 2000 onwards	89	11.8 (9.9–13.7)	73	13.9 (12.3–15.5)	4	18.0 (14.8–21.5)	161	12.8 (11.7–14.0)
Stage at diagnosis								
Early (I–IIa)	6	4.8 (2.5–7.8)	6	6.6 (3.8–10.0)	1	–	13	5.9 (4.3–7.7)
Locally advanced (IIb–IIIc)	6	33.2 (24.7–42.3)	4	35.0 (29.4–40.7)	4	35.3 (28.9–42)	13	34.5 (30.3–38.9)
Hormone receptor status								
HR+	11	9.6 (6.2–13.6)	13	10.0 (7.5–12.7)	3	13.4 (8.5–19.2)	25	9.9 (8.0–11.9)
HR−	24	15.3 (12.1–18.8)	13	22.4 (15.8–29.7)	3	40.5 (11.4–73.7)	39	17.7 (15.0–20.7)
Age group								
< 50 years	9	16.1 (10.8–22.1)	14	19.7 (15.9–23.9)	5	20.6 (15.6–26.0)	27	18.6 (15.9–21.4)
50–69 years	1	–	2	–	3	15.7 (12.4–19.3)	5	10.9 (9.0–12.9)
70+ years	1	–	0	–	2	–	3	13.3 (9.2–18.0)

^a^Studies do not always sum to the total due to exclusion of studies from the same city or hospital to avoid overlapping or duplicating when combining groups. Meta-analyses were performed when more than 3 studies were available

**Fig. 2:**
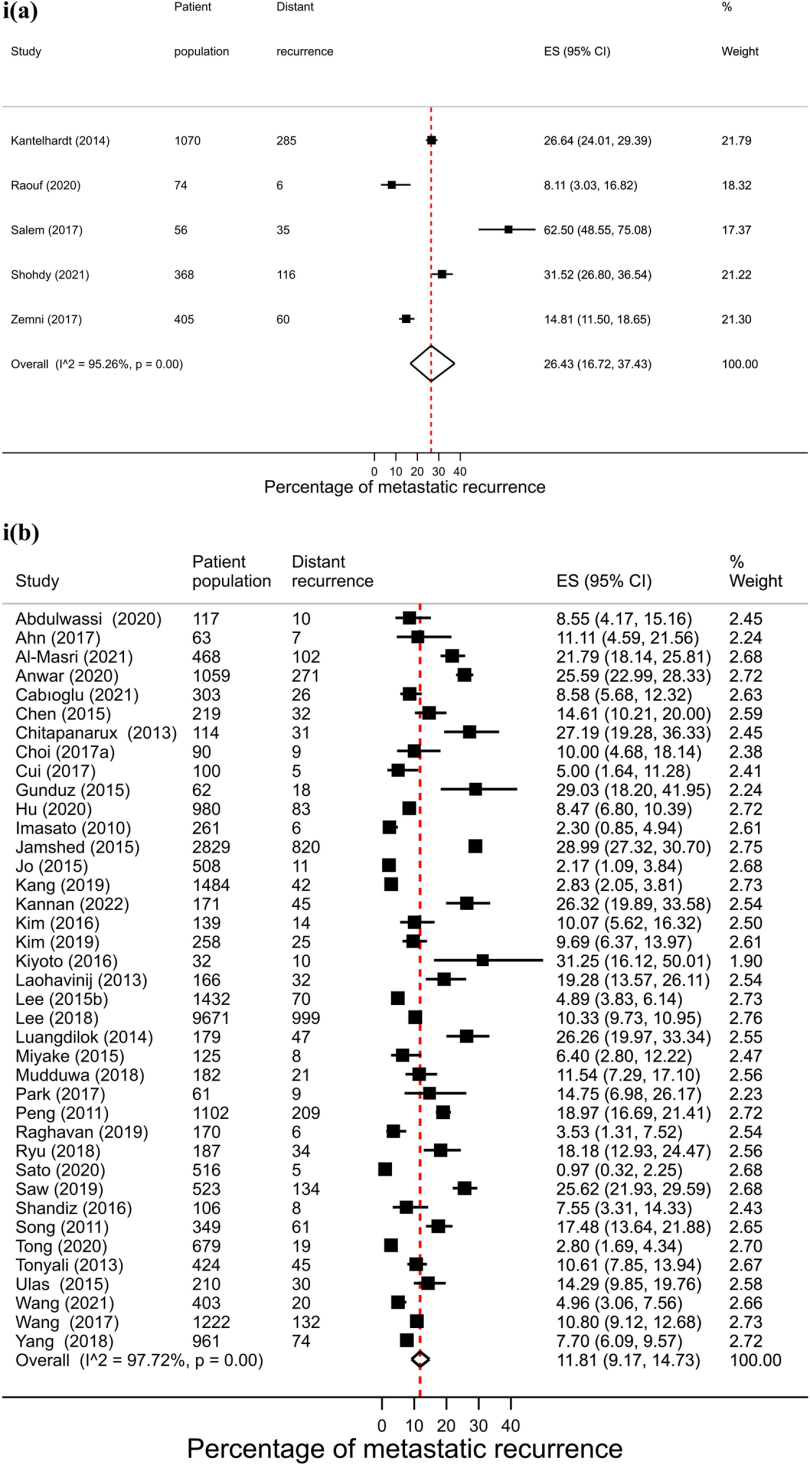

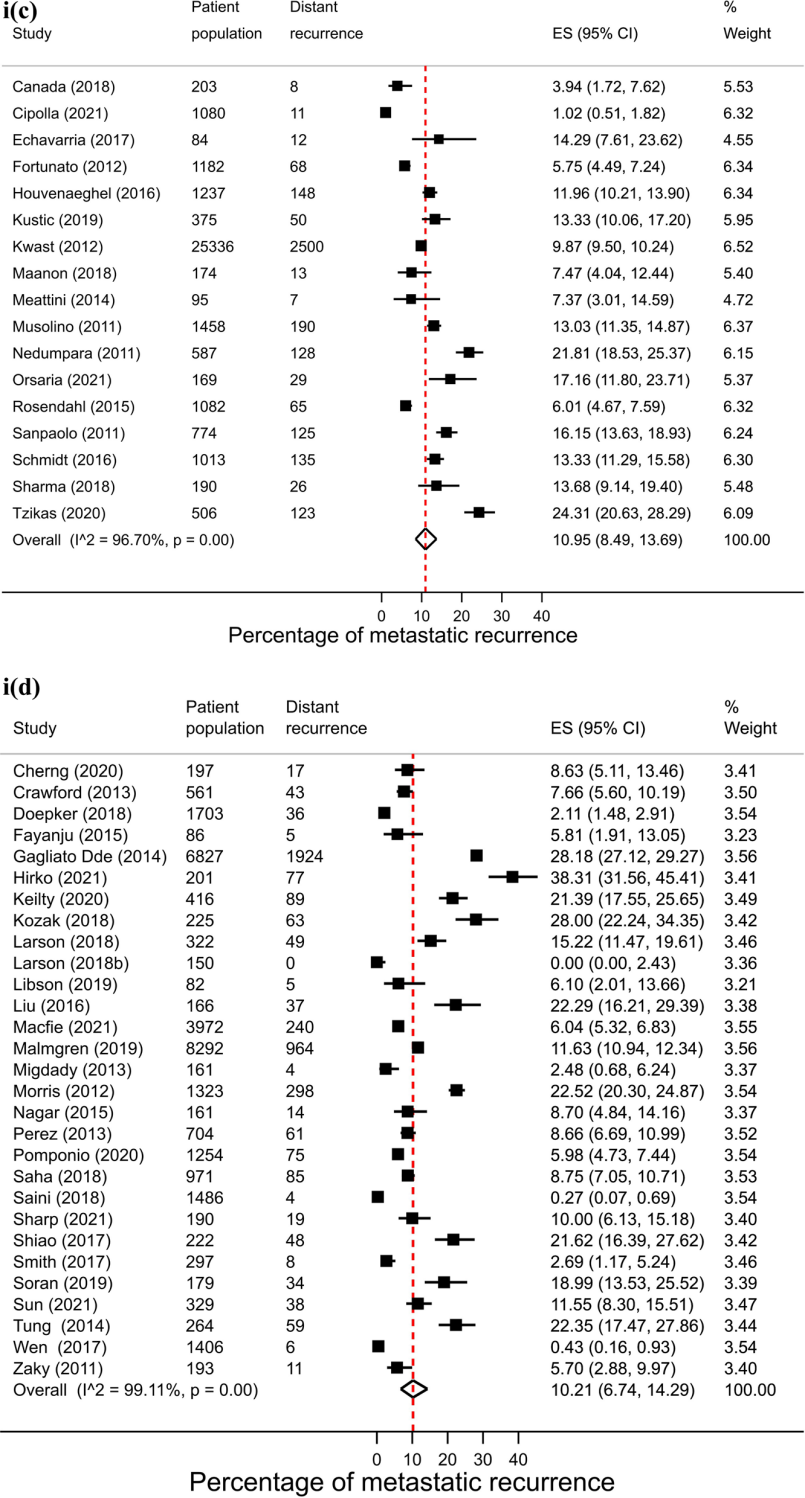

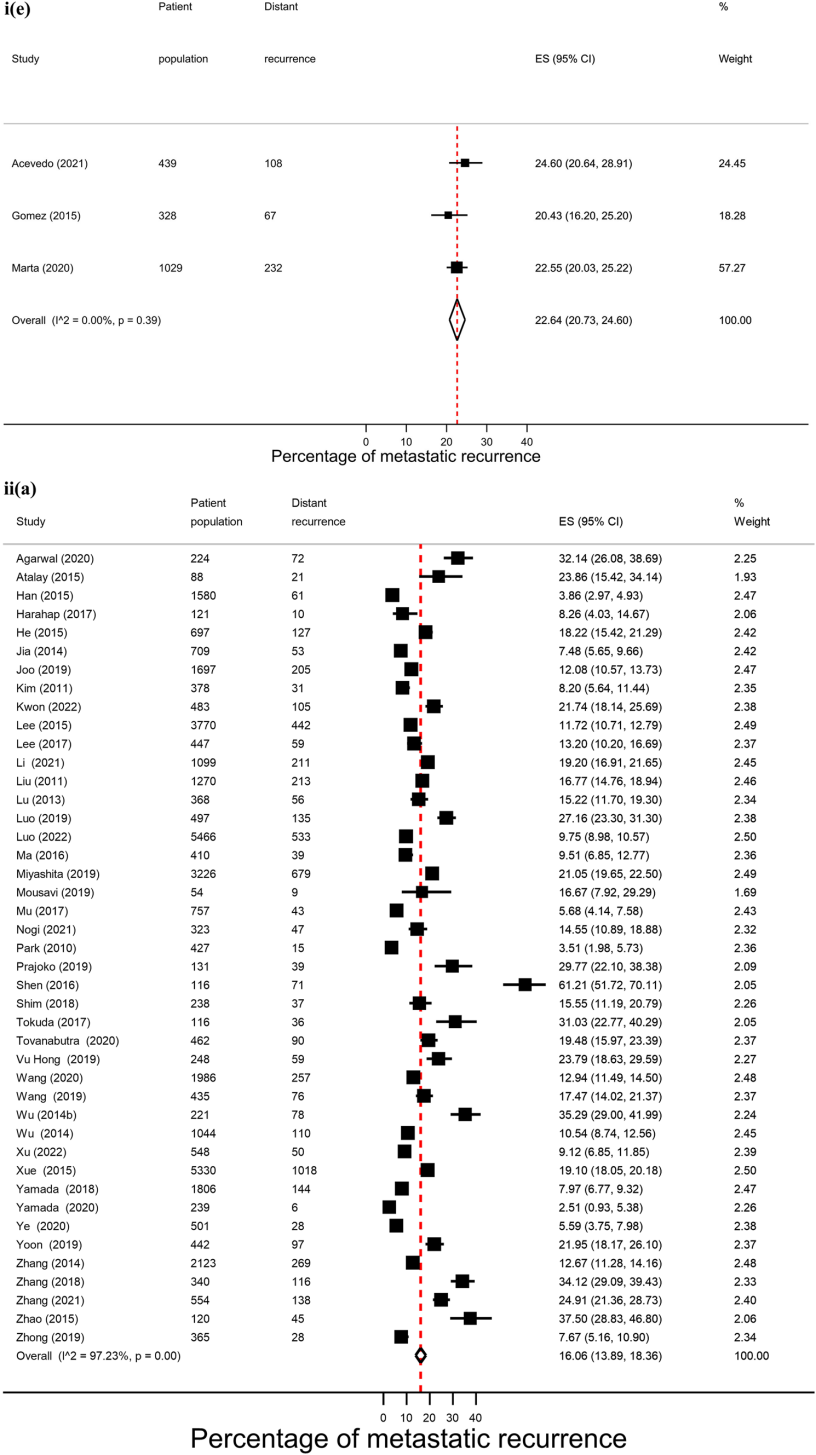

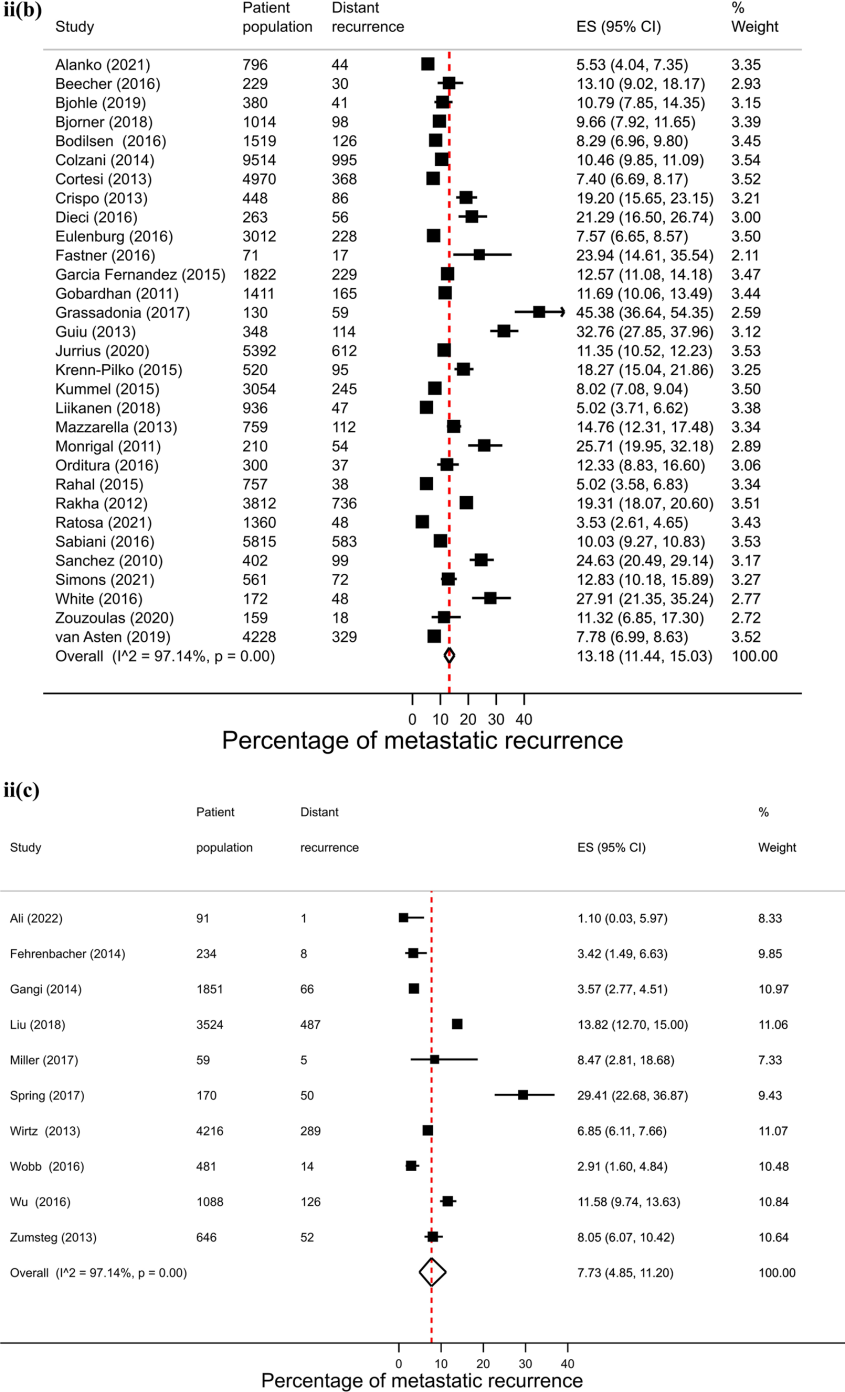

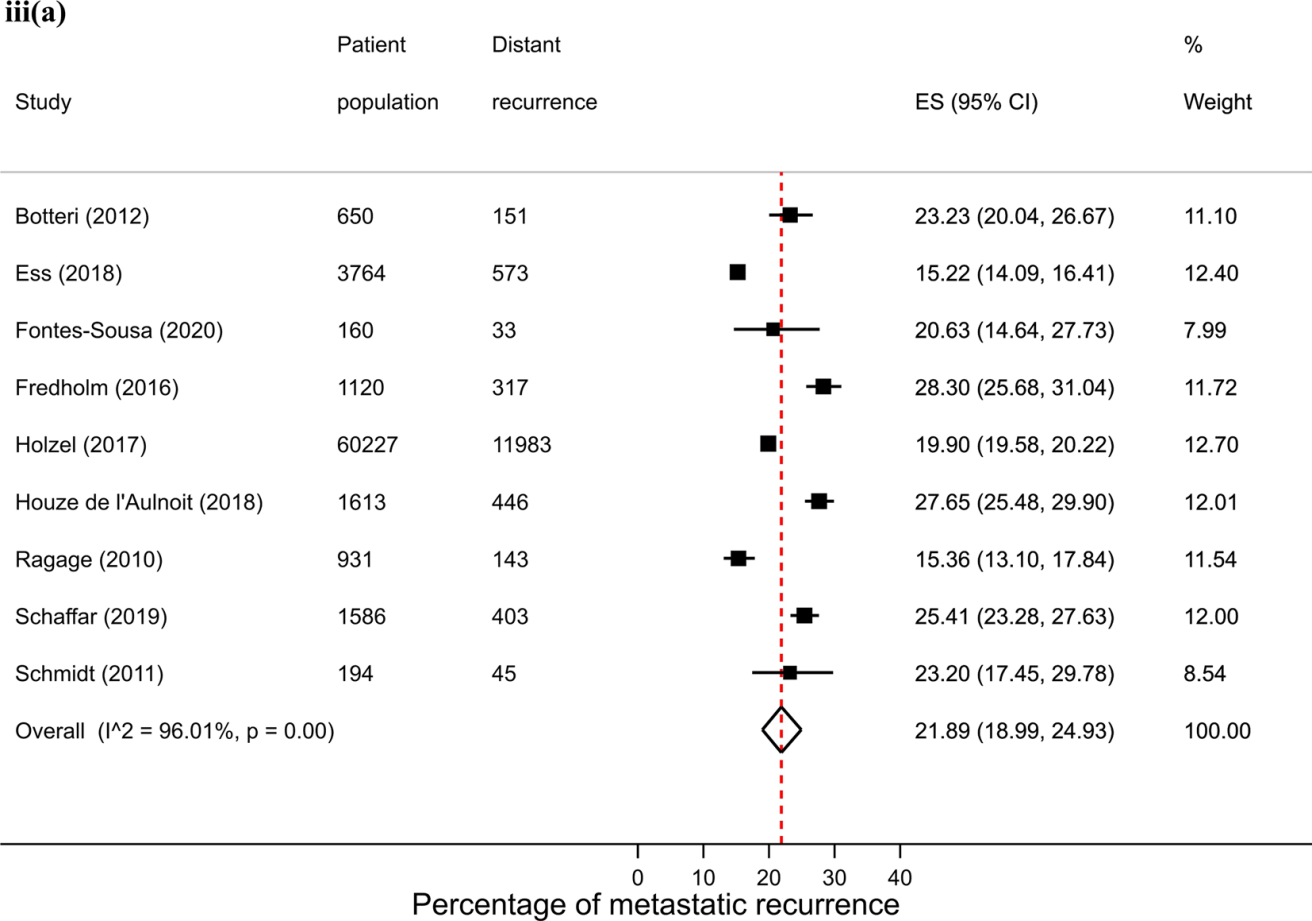
(**i**): Forest plot of the proportion of metastatic recurrences in women diagnosed with breast cancer at 1–4 years by region: **a** Africa **b** Asia **c** Europe **d** North America **e** South America. (**ii**): Forest plot of the proportion of metastatic recurrences in women diagnosed with breast cancer at 5–9 years by region: **a** Asia **b** Europe **c** North America. (**iii**): Forest plot of the proportion of metastatic recurrences in women diagnosed with breast cancer at 10+ years in Europe

Across regions, proportions of metastatic recurrence were highest in Africa followed by South America at 1–4 years median follow-up with pooled proportions of 26.4% (95% CI 16.7–37.4%) and 22.6% (95% CI 20.7–24.6%), respectively. Pooled estimates at 1–4 years median follow-up were considerably lower in other regions with estimates of 11.0% (Europe), 10.2% (North America) and 11.8% (Asia) (Fig. 2, i). Generally, proportions were higher with increasing length of median follow-up time where meta-analysis at regional level was possible. At 5–9 years median follow-up, pooled estimates were as high as 16.1% (Asia), 13.2% (Europe) and 7.7% (North America) (Fig. 2, ii). Data permitted regional pooled proportions for Europe at 10 years or more median follow-up time to be estimated: an increase to 21.9% (Fig. 2, iii).

Significant heterogeneity was observed across all meta-analyses of overall proportions of metastatic recurrence (I^2^ > 96%, *p* < 0.01).

### Calendar period of diagnosis

In analyses by calendar period of diagnosis 167 studies were included to compare metastatic recurrence proportions in patients diagnosed before 2000 and from 2000 onwards. Pooled proportions of metastatic recurrence were higher in studies that were restricted to women diagnosed before 2000 compared with those diagnosed 2000 onwards with estimates of 22.2 (95% CI 15.1–30.3%) and 12.8 (95% CI 11.7–14.0%), respectively (Table 1). Proportions increased with increasing follow-up time in studies with patients diagnosed from 2000 onwards from 11.8% at 1–4 years to 18.0% at 10+ years. Too few studies with diagnosis period before 1999 were available to permit analysis at all follow-up times, with proportions of 22.2% at 5–9 years.

### Stage at diagnosis

**Fig. 3:**
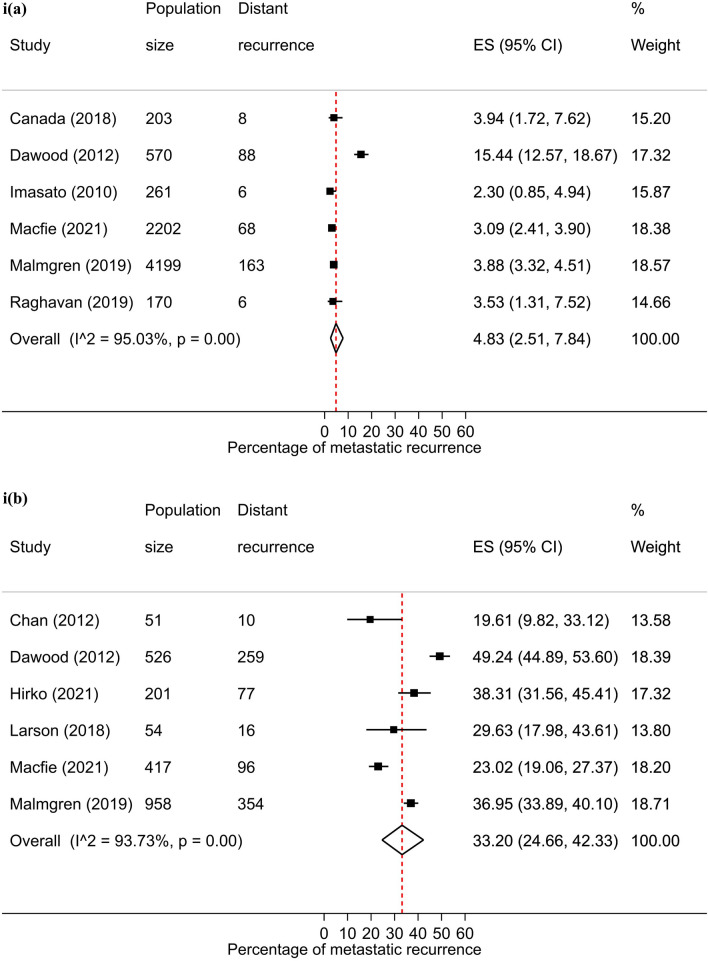

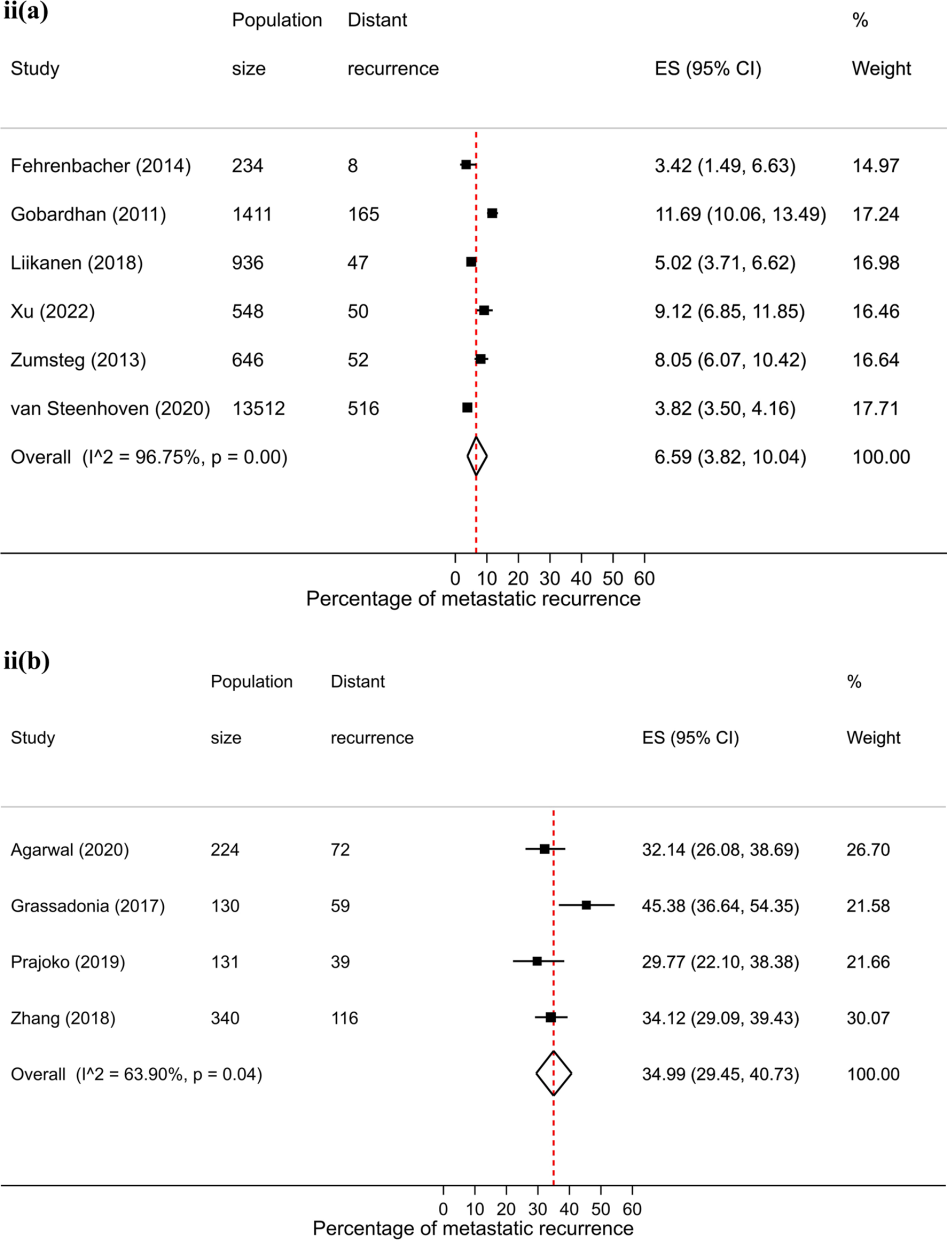

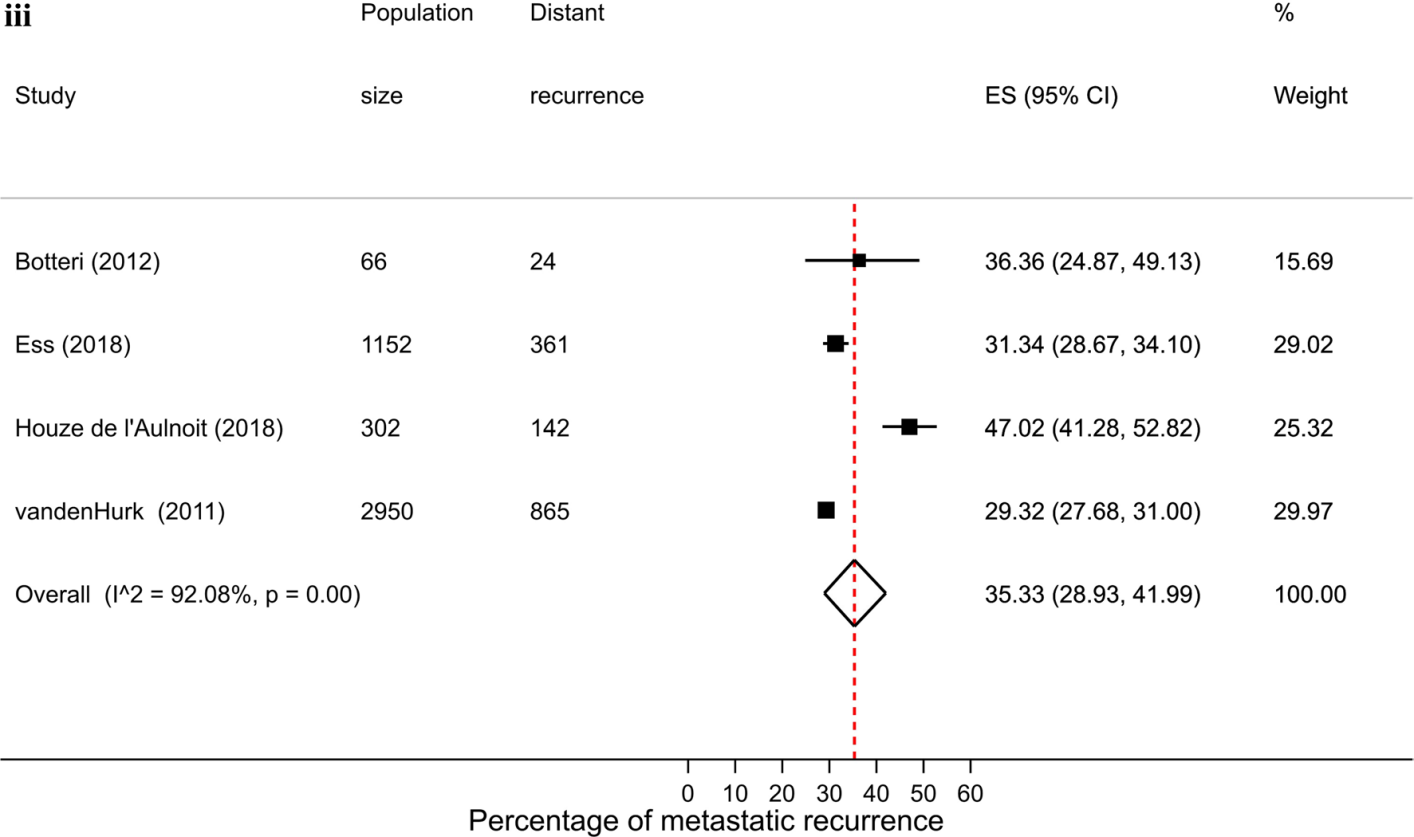
Forest plot of proportion of metastatic recurrences in women diagnosed with **a** early (I-IIa) and **b** locally advanced (IIb-IIIc) breast cancer at (**i**) 1–4 years; (**ii**) 5–9 years; (**iii**) 10+ years (locally advanced only) median follow-up

There were 26 studies that were included in the stage-specific analysis. The overall pooled proportion of metastatic recurrence in early stage (stage I-IIa) patients was 5.9% (95% CI 4.3–7.7%) and 34.5% (95% CI 30.3–38.9%) in locally advanced stage (stage IIb-IIIc) patients. Proportions of metastatic recurrence in early-stage patients increased with increasing follow-up time but were consistently higher in locally advanced patients across all median follow-time points (Fig. 3).

Substantial heterogeneity was observed in both early stage (I^2^ = 96.1%, *p* < 0.01) and locally advanced stage (I^2^ = 91.8%, *p* < 0.01) meta-analyses.

### Hormone receptor status

**Fig. 4:**
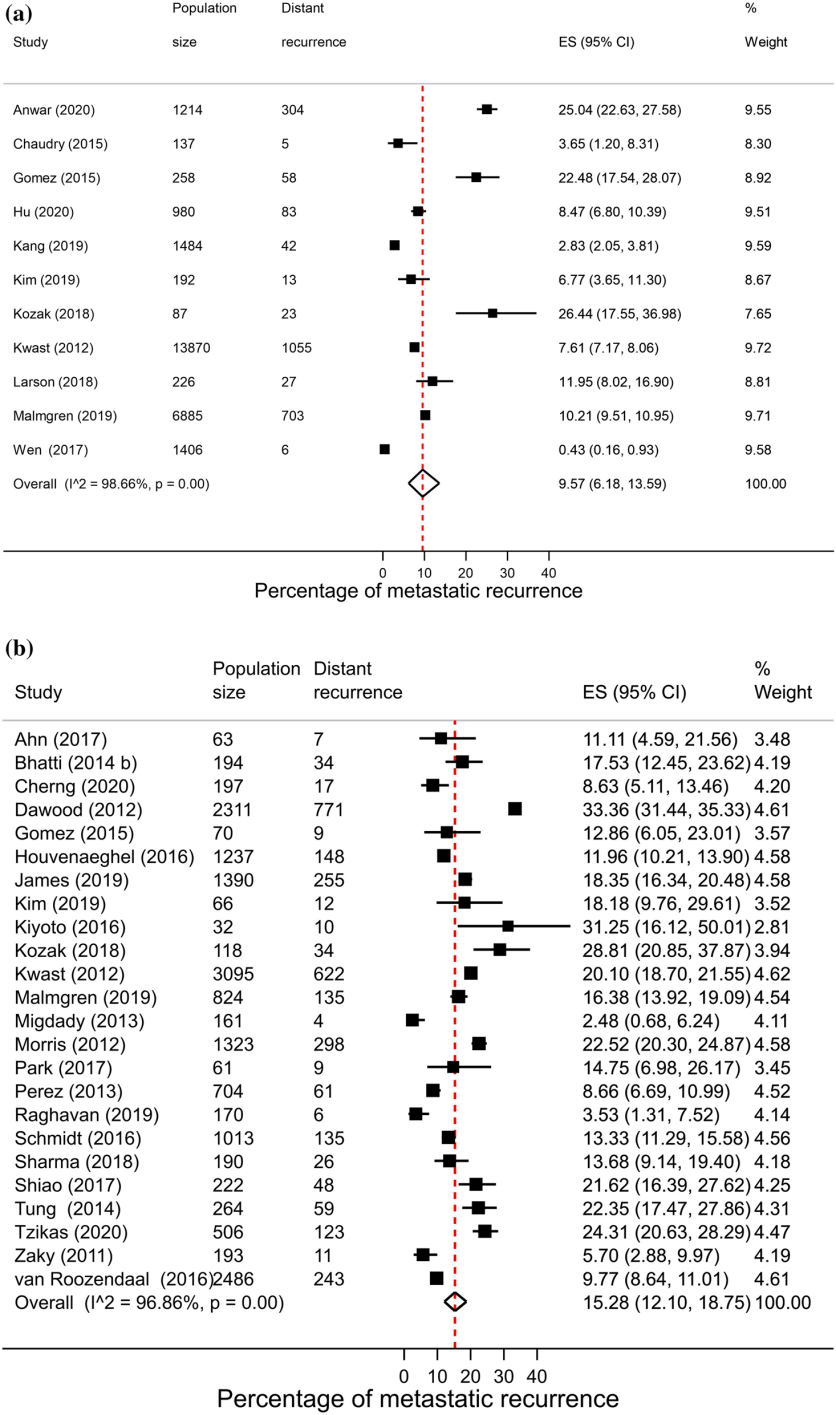

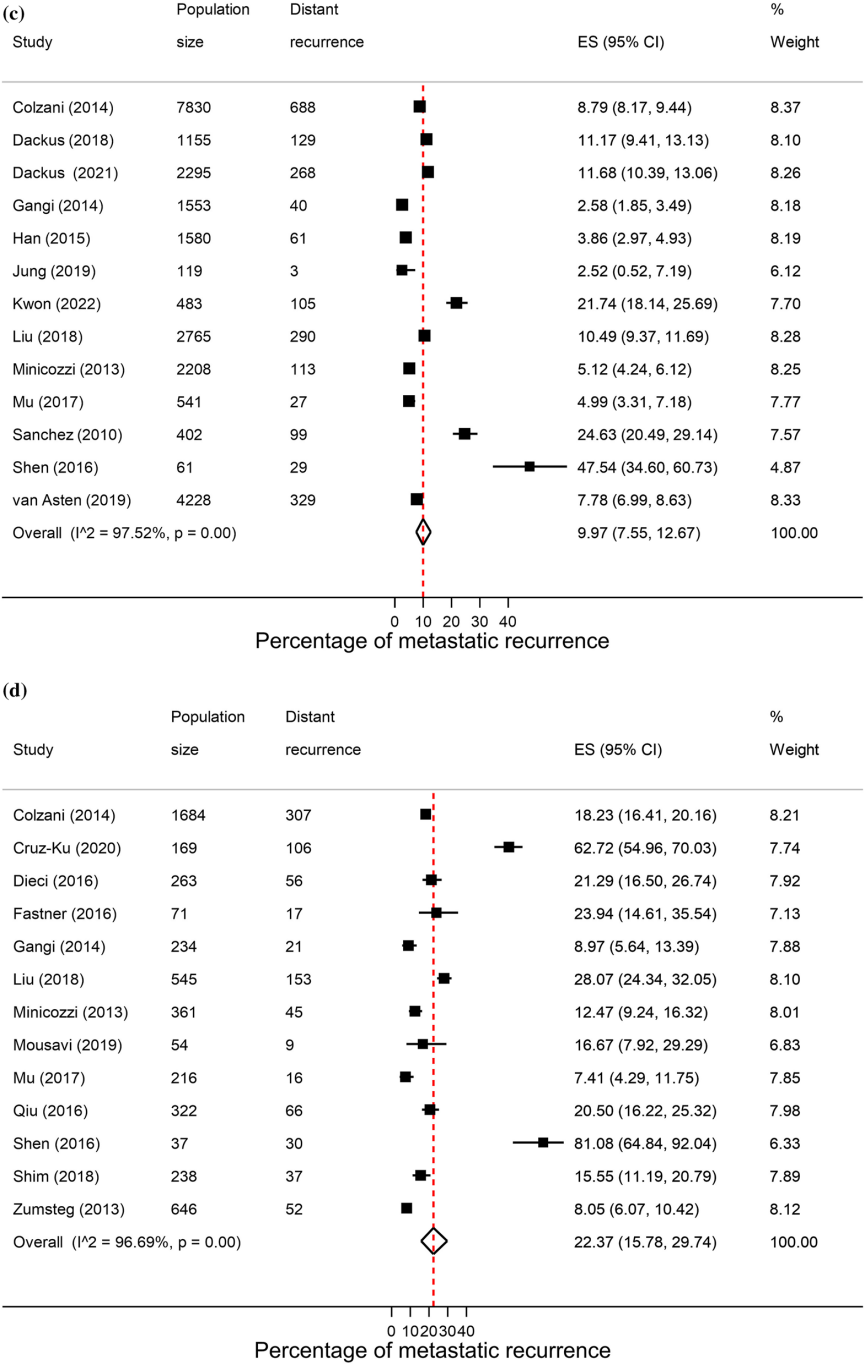

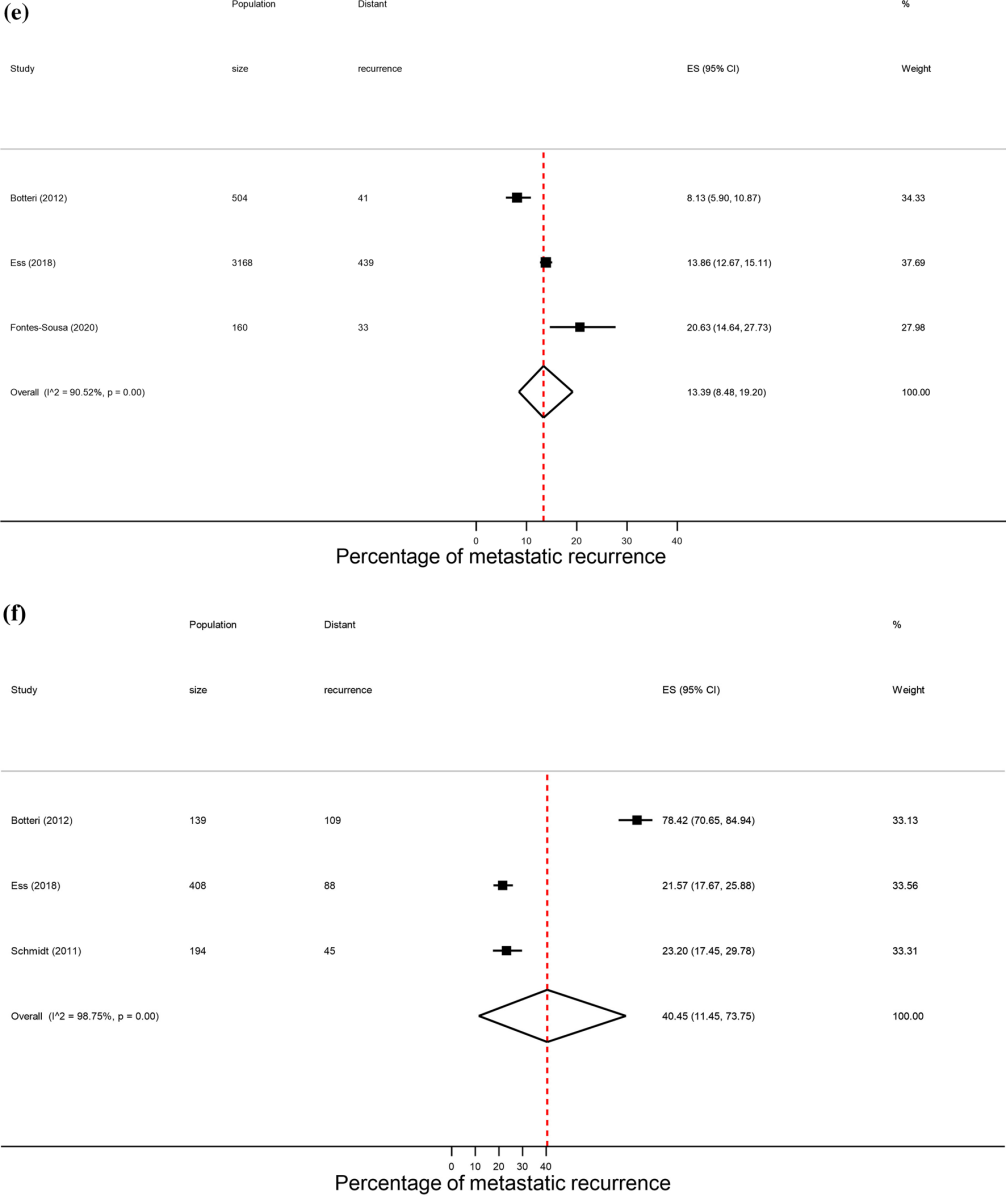
Forest plot of proportion of metastatic recurrences in women diagnosed with **a** HR+ ; **b** HR−(1–4 years median follow-up) and **c** HR+ ; **d** HR− (5–9 years median follow-up) **e** HR+ ; (f) HR− (10+ years median follow-up)

A total of 64 studies reported proportions of metastatic recurrence in either or both HR+ and HR− patients. The proportion of women experiencing metastatic recurrences was lower in HR+ patients compared to HR− patients with pooled proportions of 9.9% (8.0–11.9%) and 17.7% (95% CI 15.0–20.7%), respectively. Pooled proportions of metastatic recurrence were consistently higher in HR- patients compared to HR+ patients at 1–4 years follow-up (15.3% vs. 9.6%, respectively) and 5–9 years follow-up (22.4% vs. 10.0%). Although higher proportions were also observed at 10+ years follow-up (40.5% vs. 13.4%) these estimates were based on a small number of studies (Fig. 4).

High heterogeneity was observed in pooled analyses across both subtypes and follow-up times (I^2^ > 96.0%, *p* < 0.01).

### Age at diagnosis

**Fig. 5:**
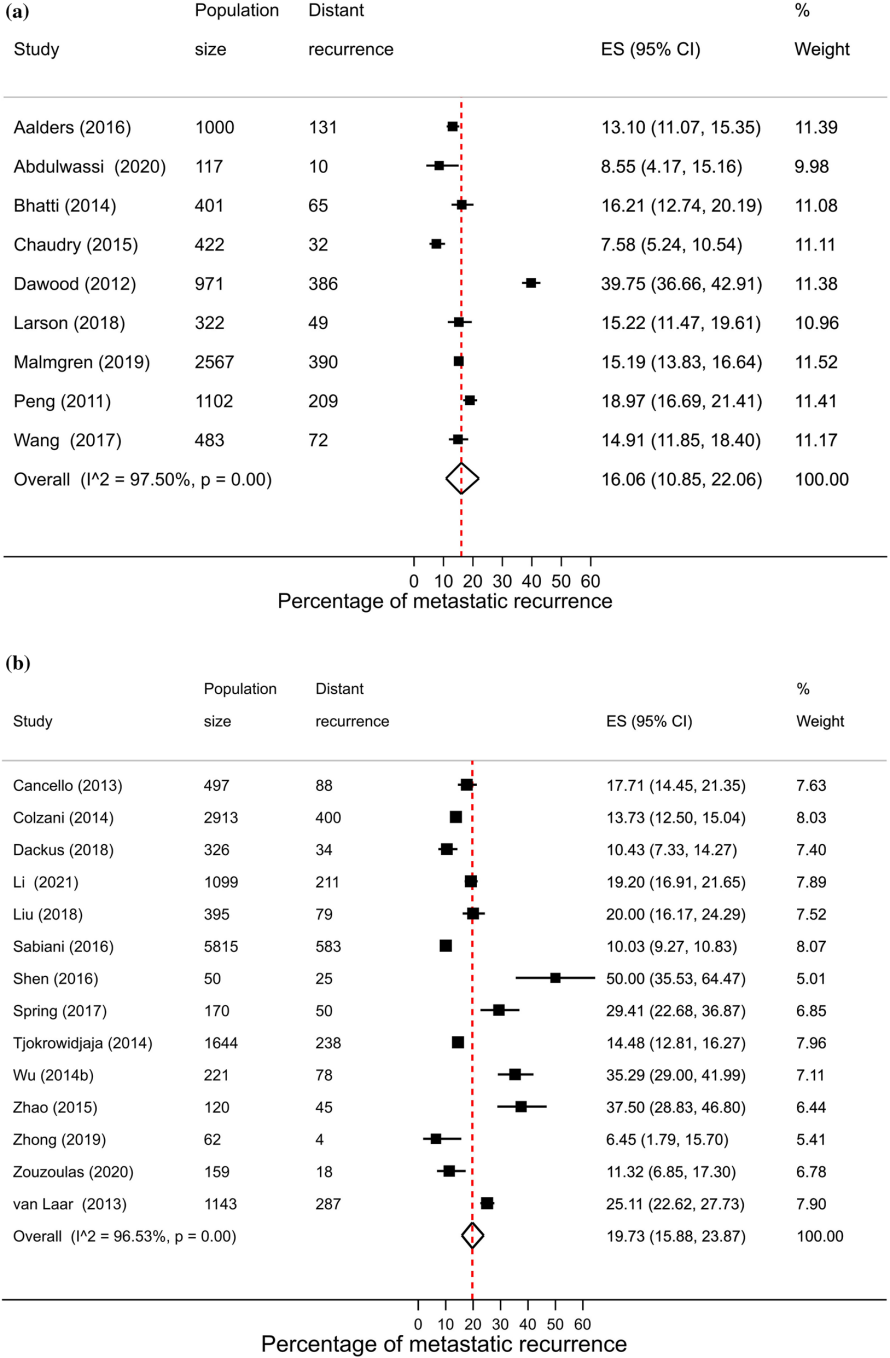

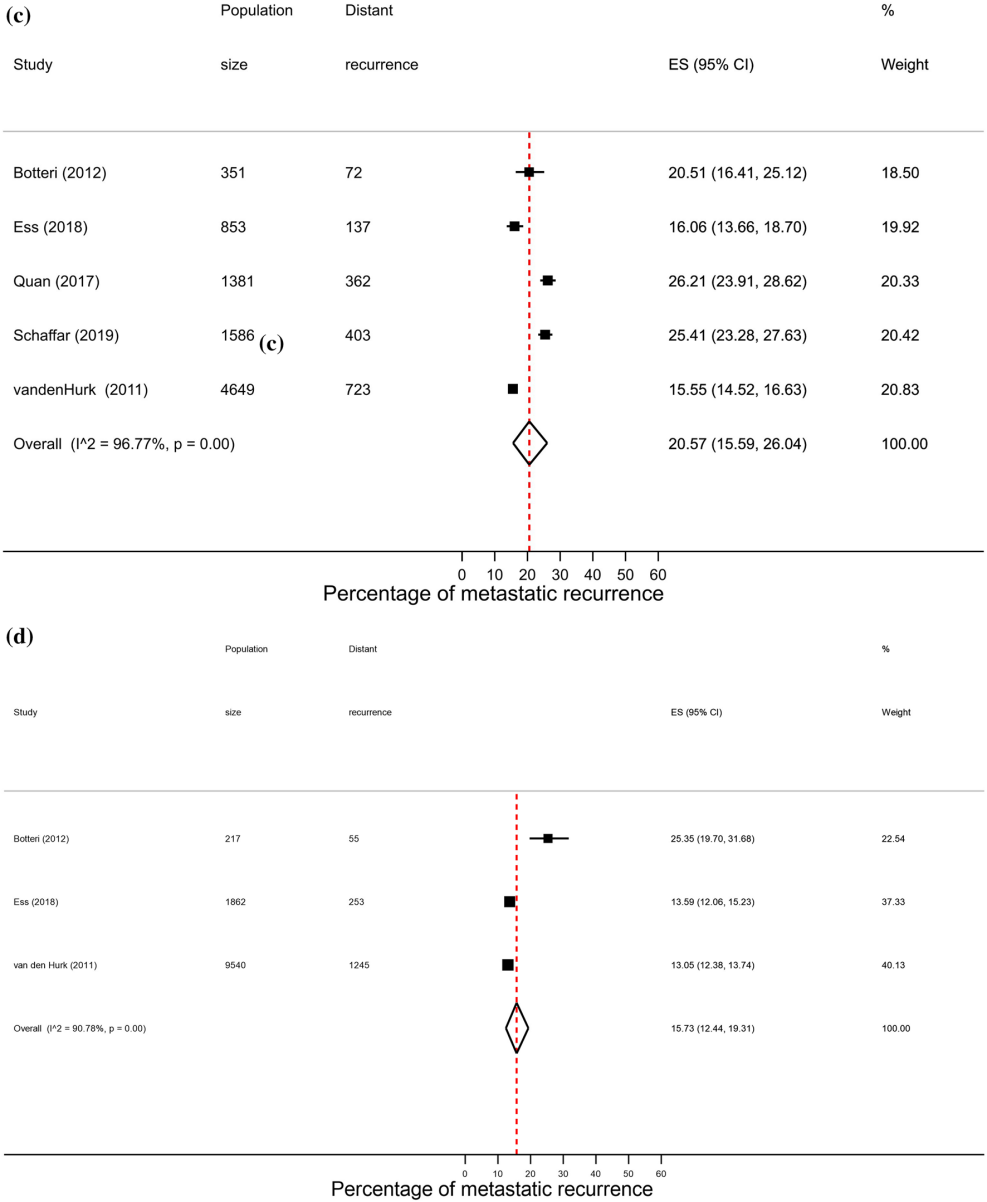
Forest plot of proportion of metastatic recurrences in women diagnosed at < 50 years (**a**–**c**) and 50–69 years (**d**) with **a** 1–4 years; **b** 5–9 years and **c**–**d** 10+ years of median follow-up

Pooled proportions of metastatic recurrence were highest in the youngest age group at initial diagnosis (< 50 years): 18.6% (95% CI 15.9–21.4%) and similar in the two older age groups (50–69 years and 70+ years) with pooled estimates of 10.9% (95% CI 9.0–12.9%) and 13.3% (95% CI 9.2–18.0%), respectively. In the youngest age group, proportions of recurrence increased with increasing follow-up time from 16.1% (95% CI 10.8–22.1%) at 1–4 years to 20.6% (95% CI 15.6–26.0%) at 10+ years. At 10+ years median follow-up, proportions of recurrence were slightly lower in 50–69 year olds compared to their younger counterpart with a pooled estimate of 15.7% (95% CI 12.4–19.3%) (Fig. 5). Too few studies were available to assess proportions of recurrence in patients diagnosed at 70+ years by median follow-up times.

### Sensitivity analyses

Sensitivity analyses restricting to 12 studies that reported recurrence proportions for both HR+ and HR− subtypes was conducted and similar results were obtained with pooled estimates of 10.4% (95% CI 8.3–12.7%) and 19.7% (95% CI 15.8–23.9%), respectively (eFigure 2 in the Supplement).

### Risk of bias

Most studies (163/217, 75.1%) showed some element of high risk of bias (eFigure 3 in the Supplement). The most common indicators of high risk of bias were related to the representativeness of the study population to the target population (42.4% of studies high risk), the likelihood of missing information on the outcome of interest (41.5% of studies high risk), if the study instrument to measure outcome parameter was shown to be reliable and valid (17.1% of studies high risk), if a random selection was used to select the sample (12.9% of studies high risk) and if the sampling frame was a representation of the target population (12.4% of studies high risk). However, similar results were obtained when restricting to studies that were of low or moderate risk of bias. Pooled proportions of metastatic recurrence ranged from 11.1% (95% CI 8.6–13.9%), 16.1% (95% CI 13.1–19.3%) and 22.0% (95% CI 14.6–30.5%) in low or moderate risk of bias studies at 1–4, 5–9 and 10+ years of median follow-up, respectively.

## Discussion

This systematic review for the first time comprehensively documents the occurrence of metastatic recurrence in women initially diagnosed with non-metastatic breast cancer, comparing across follow-up and calendar time and different data sources. Similar recurrence proportions were observed when comparing PBCR- and hospital-based studies with pooled estimates of around 13% within 5 years of diagnosis. In general, proportions of recurrence increased as median follow-up time increased, with overall proportions peaking at 23.3% after more than 10 years median follow-up. Differences were observed across world regions, with pooled estimates ranging from 11% in Europe to as high as 26% in African countries. Recurrence proportions were lower in women with early stage disease at diagnosis and with HR+ tumours compared to women diagnosed with locally advanced or HR− disease, respectively. To consider the impact of advances in treatments including targeted therapies (such as trastuzumab), the identification of subtypes and genetic testing that were implemented post-1999, recurrence patterns by calendar period of diagnosis before and after 1999 were investigated. Proportions of recurrence were higher in studies from earlier calendar period (before 2000), particularly in studies with 5–9 years median follow-up. Improvements in outcomes of patients in recent years compared with those treated in earlier era (late 1980s/early 1990s) have been reported in other studies and indicate improvements in efficacy of evidence-based treatment guidelines in recent years [8]. The lower proportions of metastatic recurrence in recent years could partly be due to reductions in loco-regional disease as a result of multidisciplinary approaches in treatment [9]. However, information on loco-regional recurrence was not investigated in the context of metastatic recurrence in this study and further investigation warranted.

The regional variation observed could be in part related to differing distributions in stage at diagnosis. with a recent study reporting much lower proportions of patients being diagnosed with early stage breast cancer in sub-Saharan African countries compared to European and US countries [10]. Such differences could partly explain the lower proportions of recurrence in some regions where early diagnosis is more common and curative treatments are more readily available. Late stage at diagnosis and limited access to adequate treatment care are factors that have been recently highlighted as needing further attention in sub-Saharan Africa countries [11, 12]. Previous studies have found that African-American women have higher risk of breast cancer recurrence than other ethnic populations [13]. Another explanation could be due to variations in the method of follow-up across studies if some studies applied a more active and closer follow-up that could lead to higher cases of metastatic recurrence detected at an earlier time point. Although an upward trend was mostly observed by increasing median follow-up time, proportions of recurrence were sometimes similar when comparing estimates of 1–4 years with 5–9 years of median follow-up with marked increases mostly observed in studies with 10+ years of follow-up. Due to fewer studies with 10+ years median follow-up, meta-analyses of this group were not always possible and future studies on recurrence with longer follow-up are needed to gain further understanding of long-term outcomes of women with breast cancer.

We noted clear differences in pooled recurrence estimates and stage at diagnosis with higher proportions occurring in women who initially presented with locally advanced disease regardless of median follow-up time, emphasising the importance of early diagnosis. The implementation of screening programmes and early detection/awareness campaigns have resulted in a stage shift in many countries allowing more opportunities for curative treatment and improved survival. Yet, advanced cancer still represents a substantial proportion of cases diagnosed in low- and middle- income countries, likely due to barriers for early detection and low awareness, as well as low accessibility to adequate diagnosis and optimal treatment options [14]. The Global Breast Cancer Initiative was launched by the WHO and international partners aiming to address the impact of breast cancer, particularly in transitioning countries. Such efforts are pivotal in increasing collaborative efforts to reduce breast cancer mortality via improved access to early diagnosis and treatment [14].

Previous studies have shown how different HR subtypes vary in their recurrence patterns, with some types recurring earlier than others [15]. Hormone receptor status has been included as a predictive factor in prediction modelling studies of distant metastases within 5 years of treatment in breast cancer patients [16].

There are several potential reasons why pooled proportions of metastatic recurrence were highest in the younger age groups. Younger women are more often diagnosed with more aggressive and faster growing tumours, more often triple negative breast cancers [17, 18], and differ in the treatment they receive from their older counterparts. Another contributing factor could be that younger women are more like to be diagnosed at a later stage (which we have also found to have higher recurrence proportions) because of delayed diagnosis due to exclusion from national screening programmes and lack of symptom awareness among younger people and clinicians. Proportions of recurrence were slightly higher in the oldest age group (70+ years) than 50–69 year olds, which could be explained by older patients falling outside of the upper age limits for screening and presenting with later stage than those participating in screening programs[10]. Other factors that could impact proportions of recurrence in the older population include differences in treatment regimens, with older cancer patients more likely to receive less aggressive treatment including chemotherapy [19, 20] or competing risks of mortality linked to comorbidities more prevalent in older age [21].

Since the 1980s, treatment of breast cancer has seen revolutionary improvements with the introduction of new chemotherapeutic agents, new categories of hormone agents, several agents targeting HER2, and multiple additional targeted therapies some of which have improved patient survival, including in some subtypes of metastatic cancer [22–24]. Considering that the number of breast cancer survivors continues to rise, in part due to improvements in treatment options, further understanding is needed of the prognostic determinants of this population.

Since the current review search was conducted, a few studies have been published and similar results were found [25, 26]. A study from Australia used cancer registry data to investigate long-term risk of distant metastases in women diagnosed with non-metastatic breast cancer and found that 22.2% of women had a distant recurrence within 14 years of follow-up, similar to the 23.3% pooled estimate observed in the current study after 10 years median follow-up [25]. Using the same data, the authors also reported that distant recurrence incidence declined over time, coinciding with the availability of new adjuvant therapies in Australia [26].

### Strengths and limitations

To our knowledge, this is the first study to systematically review the published literature of the proportion of metastatic recurrence in women diagnosed with primary non-metastatic breast cancer. We found high heterogeneity in the included studies for both overall and subgroup analyses. The heterogeneity observed is suggestive of the differences in the methods used across studies and the high risk of bias. Risk of bias was high in most studies however, sensitivity analyses restricting to low-risk studies showed similar results. Comparison across pooled results is reported on different subgroups of interest and therefore forest plots were not always based on the same group of studies, however sensitivity analysis restricting to studies that reported on both subtypes showed similar pooled estimates. To-date, the proportion of women who were diagnosed with early-stage breast cancer and later develop MBC during disease recurrence, remains largely unknown. This type of long-term follow-up information is typically not routinely collected in most cancer registries and therefore not available at the population-level. This might be improved in future as more metastases will be pathologically confirmed and easier to notify by the registries through the pathology data. We identified 35 studies from 15 PBCRs in 9 countries that investigated metastatic recurrence in the current review. With new therapies elongating survival from MBC, often for many years, it is important that women with MBC are represented in research and in surveillance at the population level.

The limitations of the studies included in this review should be noted. Information on treatment regimens were not collected therefore the impact of treatment on MBC recurrence could not be investigated. Time-to-event outcomes were not studied in this review and changes in treatment over time were not considered. Although it was not possible to calculate, for example, 10-year recurrence rates, to help ensure consistent follow-up time we focused on describing events stratified by median follow-up time which, to an extent, will account for variations in time-to-event. Moreover, a combined estimate (regardless of follow-up time from diagnosis) should be interpreted with caution due to the change in risk with longer follow-up. As few studies reported HER2 status, particularly older studies, we are unable to comment on the impact of HER2 expression on recurrence in this analysis. It is possible that differences in the follow-up of patients across studies could explain some of the variation in proportions of metastases; often the follow-up in hospital-based studies aimed to detect locoregional recurrences and not distant metastases. One could expect that a very active and close follow-up and the use of newer technologies would lead to more and earlier detection of distant metastases. However, we did not collect this information in detail. Finally, we found a lack of homogeneity of definitions or approaches in recording metastatic recurrence data, which make comparisons challenging. There is a strong need for the application of consistent definitions for registration in future studies investigating metastatic recurrence in cancer patients.

## Conclusions

In summary, this systematic review presents an overview of proportions of distant recurrence in women with initial non-metastatic breast cancer, with higher proportions particularly in those initially diagnosed under 50 years, with locally advanced disease and HR negative breast cancer.

Future population-based studies are needed to provide important insights into the prevalence of MBC to improve cancer control and allow adequate provision of services for this population. International efforts including the WHO Global Breast Cancer Initiative, The Lancet Breast Cancer Commission and the ABC Global Alliance are pivotal in improving outcomes for women with breast cancer, including metastatic breast cancer.

Population-based cancer registries should be provided definitions and guidelines to collect these data and encouraged to record recurrence information to facilitate future studies.

## Supplementary Information

Supplementary Material 1

## Data Availability

No datasets were generated or analysed during the current study.
