# Supplementary material for: Metastatic recurrence in women diagnosed with non-metastatic breast cancer: a systematic review and meta-analysis
Source: Breast Cancer Res. 2024 Nov 27;26:171. doi: 10.1186/s13058-024-01881-y (PMC11603627; doi:10.1186/s13058-024-01881-y)
Supplement: Supplementary file 1 — Supplementary Material 1 [file 13058_2024_1881_MOESM1_ESM.docx]

**eTable 1:** Search strategy

| **Concept** | **Key words, medical subject headings** |
| --- | --- |
| 1) Breast cancer | Breast neoplasms/diagnosis; breast neoplasms/epidemiology; breast neoplasms/mortality; breast neoplasms/pathology; female; humans |
| 2) Stage at diagnosis | Stage; staging AND cancer/ tumor(s); extent |
| 3) Recurrence | Recurrence; relapse; second cancer; (disease) progression; recurrent cancer; tumor recurrence; cancer invasion; metastasis; metastatic; metastases; metastatic breast cancer; relapse/recurrence; relapsed breast cancer; cancer recurrence; recurrence rate; cancer spread; neoplasm metastasis; neoplasm staging; secondary; advanced; late stage; distant |
| 4) Setting/ Registry | Cancer registry; hospital registry; hospital; institution; registry data; hospital-based cancer registry data; registries; representative; population-based |

**eTable 2:** Characteristics of included studies investigating metastatic recurrence in women diagnosed with recurrent breast cancer, ordered by country and publication date

| **World region** | **Country** | **Author (year)** | **Setting** | **Study design** | **Period of diagnosis** | **Median follow-up time** | **Population (N)** | **Patients with metastatic recurrences (N)** | **Proportion of recurrences (%)** |
| --- | --- | --- | --- | --- | --- | --- | --- | --- | --- |
| Africa | Egypt | Elsayed (2016) | Hospital | Cohort | 2005-2014 | 5-9 years | 238 | 10 | 4 |
|  | Egypt | Raouf (2020) | Hospital | Cohort | 2015-2019 | 1-4 years | 74 | 6 | 8 |
|  | Egypt | Salem (2017) | Hospital | Cohort | 2007-2014 | 1-4 years | 56 | 35 | 63 |
|  | Egypt | Shohdy (2021) | Hospital | Cohort | 2010-2015 | 1-4 years | 368 | 116 | 32 |
|  | Ethiopia | Kantelhardt (2014) | Hospital | Cohort | 2005-2010 | 1-4 years | 1070 | 285 | 27 |
|  | Tunisia | Zemni (2017) | Hospital | Cohort | 2001-2003 | 1-4 years | 405 | 60 | 15 |
| Asia | China | Chen (2015) | Hospital | Cohort | 2006-2012 | 1-4 years | 219 | 32 | 15 |
|  | China | Cui (2017) | Hospital | Cohort | 2010-2011 | 1-4 years | 100 | 5 | 5 |
|  | China | Han (2015) | Hospital | Cohort | 2005-2007 | 5-9 years | 1580 | 61 | 4 |
|  | China | He (2015) | Hospital | Cohort | 1998-2007 | 5-9 years | 697 | 127 | 18 |
|  | China | Hu (2020) | Hospital | Cohort | 2012-2016 | 1-4 years | 980 | 83 | 8 |
|  | China | Jia (2014) | Hospital | Cohort | 1999-2010 | 5-9 years | 709 | 53 | 7 |
|  | China | Li (2021) | Hospital | Cohort | 2006-2014 | 5-9 years | 1099 | 211 | 19 |
|  | China | Liu (2011) | Hospital | Cohort | 2003-2004 | 5-9 years | 1270 | 213 | 17 |
|  | China | Lu (2013) | Hospital | Cohort | 2001-2005 | 5-9 years | 368 | 56 | 15 |
|  | China | Luo (2022) | Hospital | Cohort | 2008-2016 | 5-9 years | 5466 | 533 | 10 |
|  | China | Luo (2019) | Hospital | Cohort | 2005-2013 | 5-9 years | 497 | 135 | 27 |
|  | China | Ma (2016) | Hospital | Cohort | 2008-2010 | 5-9 years | 410 | 39 | 10 |
|  | China | Mu (2017) | Hospital | Cohort | 2002-2010 | 5-9 years | 757 | 43 | 6 |
|  | China | Peng (2011) | Hospital | Case-control | 1995-2008 | 1-4 years | 1102 | 209 | 19 |
|  | China | Qiu (2016) | Hospital | Cohort | 2004-2009 | 5-9 years | 1578 | 205 | 13 |
|  | China | Shen (2016) | Hospital | Cohort | 2003-2008 | 5-9 years | 116 | 71 | 61 |
|  | China | Tong (2020) | Hospital | Cohort | 2012-2017 | 1-4 years | 679 | 19 | 3 |
|  | China | Wang (2021) | Hospital | Cohort | 2012-2016 | 1-4 years | 403 | 20 | 5 |
|  | China | Wang (2020) | Hospital | Cohort | 2000-2014 | 5-9 years | 1986 | 257 | 13 |
|  | China | Wang (2019) | Hospital | Cohort | 2011-2013 | 5-9 years | 435 | 76 | 17 |
|  | China | Wang (2017) | Hospital | Cohort | 2008-2012 | 1-4 years | 1222 | 132 | 11 |
|  | China | Wu (2014b) | Hospital | Cohort | 1998-2007 | 5-9 years | 221 | 78 | 35 |
|  | China | Wu (2014) | Hospital | Cohort | 1999-2007 | 5-9 years | 1044 | 110 | 11 |
|  | China | Xu (2022) | Hospital | Cohort | 2009-2016 | 5-9 years | 548 | 50 | 9 |
|  | China | Xue (2015) | Hospital | Cohort | 1997-2008 | 5-9 years | 5330 | 1018 | 19 |
|  | China | Yang (2018) | Hospital | Cohort | 2000-2016 | 1-4 years | 961 | 74 | 8 |
|  | China | Ye (2020) | Hospital | Cohort | 2005-2018 | 5-9 years | 501 | 28 | 6 |
|  | China | Zhang (2018) | Hospital | Cohort | 1999-2009 | 5-9 years | 340 | 116 | 34 |
|  | China | Zhang (2021) | Hospital | Cohort | 2007-2015 | 5-9 years | 554 | 138 | 25 |
|  | China | Zhang (2014) | Hospital | Cohort | 2002-2008 | 5-9 years | 2123 | 269 | 13 |
|  | China | Zhao (2015) | Hospital | Cohort | 2006-2012 | 5-9 years | 120 | 45 | 38 |
|  | China | Zhong (2019) | Hospital | Cohort | 2003-2016 | 5-9 years | 365 | 28 | 8 |
|  | India | Agarwal (2020) | Hospital | Cohort | 2004-2014 | 5-9 years | 224 | 72 | 32 |
|  | India | Kannan (2022) | Hospital | Cohort | 2014-2019 | 1-4 years | 171 | 45 | 26 |
|  | India | Raghavan (2019) | Hospital | Cohort | 2010-2011 | 1-4 years | 170 | 6 | 4 |
|  | Indonesia | Anwar (2020) | Hospital | Cohort | 2013-2018 | 1-4 years | 1059 | 271 | 26 |
|  | Indonesia | Harahap (2017) | Other | Cohort | 2010-2015 | 5-9 years | 121 | 10 | 8 |
|  | Indonesia | Prajoko (2019) | Hospital | Cohort | 2008-2011 | 5-9 years | 131 | 39 | 30 |
|  | Iran | Ghavami (2017) | Hospital | Cohort | 2001-2007 | 10+ years | 549 | 122 | 22 |
|  | Iran | Mousavi (2019) | Hospital | Cohort | 2005-2015 | 5-9 years | 54 | 9 | 17 |
|  | Iran | Shandiz (2016) | Hospital | Cohort | 2001-2008 | 1-4 years | 106 | 8 | 8 |
|  | Japan | Imasato (2010) | Hospital | Cohort | 2001-2006 | 1-4 years | 261 | 6 | 2 |
|  | Japan | Kiyoto (2016) | Hospital | Cohort | 2006-2013 | 1-4 years | 32 | 10 | 31 |
|  | Japan | Miyake (2015) | Hospital | Cohort | 2005-2011 | 1-4 years | 125 | 8 | 6 |
|  | Japan | Miyashita (2019) | Hospital | Cohort | 2004-2009 | 5-9 years | 3226 | 679 | 21 |
|  | Japan | Nogi (2021) | Hospital | Cohort | 2005-2012 | 5-9 years | 323 | 47 | 15 |
|  | Japan | Sato (2020) | Hospital | Cohort | 2008-2018 | 1-4 years | 516 | 5 | 1 |
|  | Japan | Tokuda (2017) | Hospital | Cohort | 2006-2008 | 5-9 years | 116 | 36 | 31 |
|  | Japan | Yamada (2020) | Hospital | Cohort | 2004-2014 | 5-9 years | 239 | 6 | 3 |
|  | Japan | Yamada (2018) | Hospital | Cohort | 1992-2010 | 5-9 years | 1806 | 144 | 8 |
|  | Jordan | Al-Masri (2021) | Hospital | Cohort | 2006-2014 | 1-4 years | 468 | 102 | 22 |
|  | Korea | Ahn (2017) | Hospital | Cohort | 2007-2016 | 1-4 years | 63 | 7 | 11 |
|  | Korea | Choi (2017a) | Hospital | Cohort | 2004-2013 | 1-4 years | 90 | 9 | 10 |
|  | Korea | Jo (2015) | Hospital | Cohort | 2009-2011 | 1-4 years | 508 | 11 | 2 |
|  | Korea | Joo (2019) | Hospital | Cohort | 2000-2015 | 5-9 years | 1697 | 205 | 12 |
|  | Korea | Jung (2019) | Hospital | Cohort | 1993-2012 | 5-9 years | 119 | 3 | 3 |
|  | Korea | Kang (2019) | Hospital | Cohort | 2009-2012 | 1-4 years | 1484 | 42 | 3 |
|  | Korea | Kim (2016) | Hospital | Cohort | 2009-2015 | 1-4 years | 139 | 14 | 10 |
|  | Korea | Kim (2019) | Hospital | Cohort | 2013-2014 | 1-4 years | 258 | 25 | 10 |
|  | Korea | Kim (2011) | Hospital | Cohort | 1992-2002 | 5-9 years | 378 | 31 | 8 |
|  | Korea | Kwon (2022) | Hospital | Cohort | 2003-2015 | 5-9 years | 483 | 105 | 22 |
|  | Korea | Lee (2015) | Hospital | Cohort | 2000-2008 | 5-9 years | 3770 | 442 | 12 |
|  | Korea | Lee (2017) | Hospital | Cohort | 2003-2008 | 5-9 years | 447 | 59 | 13 |
|  | Korea | Lee (2018) | Hospital | Cohort | 1993-2008 | 1-4 years | 9671 | 999 | 10 |
|  | Korea | Lee (2015b) | Hospital | Cohort | 2003-2011 | 1-4 years | 1432 | 70 | 5 |
|  | Korea | Park (2017) | Hospital | Cohort | 2010-2012 | 1-4 years | 61 | 9 | 15 |
|  | Korea | Park (2010) | Hospital | Cohort | 1994-2004 | 5-9 years | 427 | 15 | 4 |
|  | Korea | Ryu (2018) | Hospital | Cohort | 2005-2013 | 1-4 years | 187 | 34 | 18 |
|  | Korea | Shim (2018) | Hospital | Cohort | 2000-2006 | 5-9 years | 238 | 37 | 16 |
|  | Korea | Song (2011) | Hospital | Cohort | 2004-2007 | 1-4 years | 349 | 61 | 17 |
|  | Korea | Yoon (2019) | Hospital | Cohort | 1996-2014 | 5-9 years | 442 | 97 | 22 |
|  | Pakistan | Bhatti (2014 b) | Hospital | Cohort | 1997-2009 | 1-4 years | 637 | 103 | 16 |
|  | Pakistan | Bhatti (2014) | Hospital | Cohort | 1997-2009 | 1-4 years | 806 | 140 | 17 |
|  | Pakistan | Jamshed (2015) | Hospital | Cohort | 1995-2009 | 1-4 years | 2829 | 820 | 29 |
|  | Saudi Arabia | Abdulwassi  (2020) | Hospital | Cohort | 2009-2017 | 1-4 years | 117 | 10 | 9 |
|  | Singapore | Saw (2019) | Hospital | Cohort | 2000-2014 | 1-4 years | 523 | 134 | 26 |
|  | Sri Lanka | Mudduwa (2018) | Hospital | Cohort | 2014-2016 | 1-4 years | 182 | 21 | 12 |
|  | Thailand | Chitapanarux  (2013) | Hospital | Cohort | 2004-2007 | 1-4 years | 114 | 31 | 27 |
|  | Thailand | Laohavinij (2013) | Hospital | Cohort | 1998-2007 | 1-4 years | 166 | 32 | 19 |
|  | Thailand | Luangdilok (2014) | Hospital | Cohort | 2007-2011 | 1-4 years | 179 | 47 | 26 |
|  | Thailand | Tovanabutra (2020) | Hospital | Cohort | 2012-2014 | 5-9 years | 462 | 90 | 19 |
|  | Turkey | Atalay (2015) | Hospital | Cohort | 2002-2003 | 5-9 years | 88 | 21 | 24 |
|  | Turkey | Cabıoglu (2021) | Hospital | Cohort | 2004-2018 | 1-4 years | 303 | 26 | 9 |
|  | Turkey | Duraker (2020) | Hospital | Cohort | 1993-2002 | 10+ years | 3280 | 1164 | 35 |
|  | Turkey | Gunduz (2015) | Hospital | Cohort | 2008-2010 | 1-4 years | 62 | 18 | 29 |
|  | Turkey | Tonyali (2013) | Hospital | Cohort | 2007-2011 | 1-4 years | 424 | 45 | 11 |
|  | Turkey | Ulas (2015) | Hospital | Cohort | 2009-2014 | 1-4 years | 210 | 30 | 14 |
|  | Vietnam | Vu Hong (2019) | Hospital | Cohort | 2002-2003 | 5-9 years | 248 | 59 | 24 |
| Europe | Austria | Fastner (2016) | Hospital | Cohort | 1998-2005 | 5-9 years | 71 | 17 | 24 |
|  | Austria | Krenn-Pilko (2015) | Hospital | Cohort | 1999-2004 | 5-9 years | 520 | 95 | 18 |
|  | Belgium | van Asten (2019) | Hospital | Cohort | 2000-2012 | 5-9 years | 4228 | 329 | 8 |
|  | Croatia | Kustic (2019) | Hospital | Cohort | 2007-2013 | 1-4 years | 375 | 50 | 13 |
|  | Denmark | Bodilsen  (2016) | PBCR | Cohort | 2000-2009 | 5-9 years | 1519 | 126 | 8 |
|  | EU | Mazzarella (2013) | Hospital | Cohort | 1995-2005 | 5-9 years | 759 | 112 | 15 |
|  | Finland | Alanko (2021) | Other | Cohort | 2006-2014 | 5-9 years | 796 | 44 | 6 |
|  | Finland | Liikanen (2018) | Hospital | Cohort | 2001-2005 | 5-9 years | 936 | 47 | 5 |
|  | France | Guiu (2013) | Hospital | Cohort | 1978-2008 | 5-9 years | 348 | 114 | 33 |
|  | France | Houvenaeghel (2016) | Hospital | Cohort | 1987-2011 | 1-4 years | 1237 | 148 | 12 |
|  | France | Houze de l'Aulnoit (2018) | Hospital | Cohort | 1977-2013 | 10+ years | 1613 | 446 | 28 |
|  | France | Monrigal (2011) | Hospital | Cohort | 1990-2008 | 5-9 years | 210 | 54 | 26 |
|  | France | Ragage (2010) | Hospital | Cohort | 1989-1992 | 10+ years | 931 | 143 | 15 |
|  | France | Rahal (2015) | Hospital | Cohort | 1998-2008 | 5-9 years | 757 | 38 | 5 |
|  | France | Rossi (2015) | Hospital | Cohort | 1981-2008 | 5-9 years | 32502 | 5946 | 18 |
|  | France | Sabiani (2016) | Hospital | Cohort | 1980-2014 | 5-9 years | 5815 | 583 | 10 |
|  | Germany | Eulenburg (2016) | Other | Cohort | 2001-2005 | 5-9 years | 3012 | 228 | 8 |
|  | Germany | Holzel (2017) | PBCR | Cohort | 1978-2013 | 10+ years | 60227 | 11983 | 20 |
|  | Germany | Jueckstock (2015) | Hospital | Cohort | 1963-2003 | 5-9 years | 131 | 28 | 21 |
|  | Germany | Kummel (2015) | Hospital | Cohort | 1998-2011 | 5-9 years | 3054 | 245 | 8 |
|  | Germany | Schmidt (2016) | Hospital | Cohort | 2002-2015 | 1-4 years | 1013 | 135 | 13 |
|  | Germany | Schmidt (2011) | Hospital | Cohort | 1986-2000 | 10+ years | 194 | 45 | 23 |
|  | Germany | van den Hurk (2011) | PBCR | Cohort | 1978-2003 | 10+ years | 33771 | 5490 | 16 |
|  | Greece | Zouzoulas (2020) | Hospital | Cohort | 2003-2016 | 5-9 years | 159 | 18 | 11 |
|  | Ireland | Beecher (2016) | Hospital | Cohort | 2004-2009 | 5-9 years | 229 | 30 | 13 |
|  | Italy | Botteri (2012) | Other | Cohort | 1997-2001 | 10+ years | 650 | 151 | 23 |
|  | Italy | Cancello (2013) | Other | Cohort | 1995-2006 | 5-9 years | 497 | 88 | 18 |
|  | Italy | Cipolla (2021) | Hospital | Cohort | 2013-2019 | 1-4 years | 1080 | 11 | 1 |
|  | Italy | Cortesi (2013) | PBCR | Cohort | 1997-2007 | 5-9 years | 4970 | 368 | 7 |
|  | Italy | Crispo (2013) | Other | Case series | 2004-2006 | 5-9 years | 448 | 86 | 19 |
|  | Italy | Dieci (2016) | Hospital | Cohort | 2000-2015 | 5-9 years | 263 | 56 | 21 |
|  | Italy | Fortunato (2012) | Hospital | Cohort | 2000-2010 | 1-4 years | 1182 | 68 | 6 |
|  | Italy | Grassadonia (2017) | Hospital | Cohort | 1990-2015 | 5-9 years | 130 | 59 | 45 |
|  | Italy | Meattini (2014) | Hospital | Cohort | 2003-2012 | 1-4 years | 95 | 7 | 7 |
|  | Italy | Minicozzi (2013) | PBCR | Cohort | 2003-2005 | 5-9 years | 3203 | 225 | 7 |
|  | Italy | Musolino (2011) | PBCR | Cohort | 2004-2007 | 1-4 years | 1458 | 190 | 13 |
|  | Italy | Orditura (2016) | Hospital | Cohort | 1999-2015 | 5-9 years | 300 | 37 | 12 |
|  | Italy | Orsaria (2021) | Hospital | Case series | 2004-2020 | 1-4 years | 169 | 29 | 17 |
|  | Italy | Sanpaolo (2011) | Hospital | Cohort | 2000-2008 | 1-4 years | 774 | 125 | 16 |
|  | Italy | Sanpaolo (2012) | Hospital | Cohort | 2000-2006 | 5-9 years | 195 | 20 | 10 |
|  | Netherlands | Aalders (2016) | PBCR | Cohort | 2003-2008 | 1-4 years | 1000 | 131 | 13 |
|  | Netherlands | Barele (2020) | Hospital | Cohort | 1990-2014 | 5-9 years | 386 | 46 | 12 |
|  | Netherlands | Dackus (2018) | PBCR | Cohort | 2005-2007 | 5-9 years | 1155 | 129 | 11 |
|  | Netherlands | Dackus (2021) | PBCR | Cohort | 2004-2007 | 5-9 years | 2295 | 268 | 12 |
|  | Netherlands | Gobardhan (2011) | Hospital | Cohort | 2000-2003 | 5-9 years | 1411 | 165 | 12 |
|  | Netherlands | Kwast (2012) | PBCR | Cohort | 2003-2005 | 1-4 years | 25336 | 2500 | 10 |
|  | Netherlands | Simons (2021) | Hospital | Cohort | 2008-2017 | 5-9 years | 561 | 72 | 13 |
|  | Netherlands | van Laar (2013) | PBCR | Cohort | 1988-2010 | 5-9 years | 1143 | 287 | 25 |
|  | Netherlands | van Roozendaal (2016) | PBCR | Cohort | 2005-2008 | 1-4 years | 2486 | 243 | 10 |
|  | Netherlands | van Steenhoven (2020) | PBCR | Cohort | 2005-2008 | 5-9 years | 13512 | 516 | 4 |
|  | Portugal | Fontes-Sousa (2020) | Hospital | Cohort | 1995-2002 | 10+ years | 160 | 33 | 21 |
|  | Slovenia | Ratosa (2021) | Hospital | Cohort | 2001-2013 | 5-9 years | 1360 | 48 | 4 |
|  | Spain | Canada (2018) | Hospital | Cohort | 2006-2015 | 1-4 years | 203 | 8 | 4 |
|  | Spain | Garcia Fernandez (2015) | Hospital | Cohort | 2005 | 5-9 years | 1822 | 229 | 13 |
|  | Spain | Maanon (2018) | Hospital | Cohort | 2009-2012 | 1-4 years | 174 | 13 | 7 |
|  | Spain | Echavarria (2017) | Hospital | Cohort | 2008-2015 | 1-4 years | 84 | 12 | 14 |
|  | Sweden | Bjohle (2019) | Hospital | Cohort | 2009-2011 | 5-9 years | 380 | 41 | 11 |
|  | Sweden | Bjorner (2018) | Hospital | Cohort | 2002-2012 | 5-9 years | 1014 | 98 | 10 |
|  | Sweden | Colzani (2014) | PBCR | Cohort | 1990-2006 | 5-9 years | 9514 | 995 | 10 |
|  | Sweden | Fredholm (2016) | PBCR | Cohort | 1992-2005 | 10+ years | 1120 | 317 | 28 |
|  | Sweden | Rosendahl (2015) | Hospital | Cohort | 2002-2012 | 1-4 years | 1082 | 65 | 6 |
|  | Sweden | Sanchez (2010) | Hospital | Cohort | 1991-1996 | 5-9 years | 402 | 99 | 25 |
|  | Sweden | Tzikas (2020) | PBCR | Cohort | 2007-2015 | 1-4 years | 506 | 123 | 24 |
|  | Switzerland | Ess (2018) | PBCR | Cohort | 2003-2005 | 10+ years | 3764 | 573 | 15 |
|  | Switzerland | Schaffar (2019) | PBCR | Cohort | 1970-2012 | 10+ years | 1586 | 403 | 25 |
|  | UK | Jurrius (2020) | Hospital | Cohort | 1975-2006 | 5-9 years | 5392 | 612 | 11 |
|  | UK | Nedumpara (2011) | Hospital | Cohort | 1996-2007 | 1-4 years | 587 | 128 | 22 |
|  | UK | Purushotham (2014) | Hospital | Cohort | 1986-2006 | 5-9 years | 3553 | 736 | 21 |
|  | UK | Rakha (2012) | Hospital | Cohort | 1989-2004 | 5-9 years | 3812 | 736 | 19 |
|  | UK | White (2016) | Hospital | Cohort | 1994-2013 | 5-9 years | 172 | 48 | 28 |
| North America | USA & Spain | Sharma (2018) | Hospital | Cohort | 2010-2015 | 1-4 years | 190 | 26 | 14 |
|  | Canada | Ali (2022) | PBCR | Cohort | 2008-2017 | 5-9 years | 91 | 1 | 1 |
|  | Canada | Keilty (2020) | Hospital | Cohort | 2008-2015 | 1-4 years | 416 | 89 | 21 |
|  | Canada | Quan (2017) | PBCR | Cohort | 1994-2003 | 10+ years | 1381 | 362 | 26 |
|  | Canada | Sopik (2019) | Hospital | Cohort | 1987-2000 | 10+ years | 2312 | 523 | 23 |
|  | Canada | Wu (2016) | Hospital | Cohort | 2004-2012 | 5-9 years | 1088 | 126 | 12 |
|  | US | Chaudry (2015) | Hospital | Cohort | 1988-2009 | 1-4 years | 749 | 44 | 6 |
|  | US | Cherng (2020) | Hospital | Cohort | 2005-2015 | 1-4 years | 197 | 17 | 9 |
|  | US | Crawford (2013) | Hospital | Cohort | 2000-2010 | 1-4 years | 561 | 43 | 8 |
|  | US | Dawood (2012) | Hospital | Cohort | 1990-2010 | 1-4 years | 2311 | 771 | 33 |
|  | US | Doepker (2018) | PBCR | Cohort | 1999-2015 | 1-4 years | 1703 | 36 | 2 |
|  | US | Fayanju (2015) | Hospital | Cohort | 1999-2010 | 1-4 years | 86 | 5 | 6 |
|  | US | Fehrenbacher (2014) | Other | Cohort | 2000-2006 | 5-9 years | 234 | 8 | 3 |
|  | US | Gagliato Dde (2014) | Hospital | Cohort | 1997-2011 | 1-4 years | 6827 | 1924 | 28 |
|  | US | Gangi (2014) | Hospital | Cohort | 2000-2012 | 5-9 years | 1851 | 66 | 4 |
|  | US | Hirko (2021) | Hospital | Cohort | 1997-2019 | 1-4 years | 201 | 77 | 38 |
|  | US | Kozak (2018) | Hospital | Cohort | 2005-2015 | 1-4 years | 225 | 63 | 28 |
|  | US | Larson (2018b) | Hospital | Cohort | 2011-2017 | 1-4 years | 150 | 0 | 0 |
|  | US | Larson (2018) | Hospital | Cohort | 2006-2013 | 1-4 years | 322 | 49 | 15 |
|  | US | Libson (2019) | Hospital | Cohort | 2005-2008 | 1-4 years | 82 | 5 | 6 |
|  | US | Liu (2016) | Hospital | Cohort | 2000-2013 | 1-4 years | 166 | 37 | 22 |
|  | US | Liu (2018) | Hospital | Cohort | 2000-2016 | 5-9 years | 3524 | 487 | 14 |
|  | US | Macfie (2021) | Hospital | Cohort | 2002-2011 | 1-4 years | 3972 | 240 | 6 |
|  | US | Malmgren (2019) | Hospital | Cohort | 1990-2011 | 1-4 years | 8292 | 964 | 12 |
|  | US | Migdady (2013) | Hospital | Cohort | 2000-2010 | 1-4 years | 161 | 4 | 2 |
|  | US | Miller (2017) | Hospital | Cohort | 2005-2010 | 5-9 years | 59 | 5 | 8 |
|  | US | Morris (2012) | Hospital | Cohort | 1998-2007 | 1-4 years | 1323 | 298 | 23 |
|  | US | Nagar (2015) | Hospital | Cohort | 2003-2010 | 1-4 years | 161 | 14 | 9 |
|  | US | Perez (2013) | Hospital | Cohort | 1999-2007 | 1-4 years | 704 | 61 | 9 |
|  | US | Pomponio (2020) | Hospital | Cohort | 2009-2018 | 1-4 years | 1254 | 75 | 6 |
|  | US | Saha (2018) | Hospital | Cohort | 2000-2014 | 1-4 years | 971 | 85 | 9 |
|  | US | Saini (2018) | Hospital | Cohort | 2002-2014 | 1-4 years | 1486 | 4 | 0 |
|  | US | Sharp (2021) | Hospital | Cohort | 2004-2018 | 1-4 years | 190 | 19 | 10 |
|  | US | Shiao (2017) | Hospital | Cohort | 1998-2016 | 1-4 years | 222 | 48 | 22 |
|  | US | Smith (2017) | Hospital | Cohort | 2007-2016 | 1-4 years | 297 | 8 | 3 |
|  | US | Soran (2019) | Hospital | Cohort | 2009-2014 | 1-4 years | 179 | 34 | 19 |
|  | US | Spring (2017) | Hospital | Cohort | 1998-2014 | 5-9 years | 170 | 50 | 29 |
|  | US | Sun (2021) | Hospital | Cohort | 1999-2018 | 1-4 years | 329 | 38 | 12 |
|  | US | Tung (2014) | Hospital | Cohort | 1996-2004 | 1-4 years | 264 | 59 | 22 |
|  | US | Wen (2017) | Hospital | Cohort | 2008-2013 | 1-4 years | 1406 | 6 | 0 |
|  | US | Wirtz (2013) | Other | Cohort | 1990-2008 | 5-9 years | 4216 | 289 | 7 |
|  | US | Wobb (2016) | Hospital | Cohort | 1993-2011 | 5-9 years | 481 | 14 | 3 |
|  | US | Zaky (2011) | Hospital | Cohort | 2003-2004 | 1-4 years | 193 | 11 | 6 |
|  | US | Zumsteg (2013) | Hospital | Cohort | 1999-2008 | 5-9 years | 646 | 52 | 8 |
| South America | Brazil | Marta (2020) | Hospital | Cohort | 2008-2014 | 1-4 years | 1029 | 232 | 23 |
|  | Chile | Acevedo (2021) | Hospital | Cohort | 2009-2019 | 1-4 years | 439 | 108 | 25 |
|  | Colombia | Gomez (2015) | Hospital | Cohort | 2009-2011 | 1-4 years | 328 | 67 | 20 |
|  | Peru | Cruz-Ku (2020) | Hospital | Cohort | 2000-2014 | 5-9 years | 169 | 106 | 63 |
| Oceania | Australia | Chan (2012) | Hospital | Cohort | 2005-2006 | 1-4 years | 51 | 10 | 20 |
|  | Australia | Morley (2010) | Other | Case-control | 1991-1998 | 5-9 years | 1029 | 267 | 26 |
|  | Australia | Naher (2018) | Hospital | Cohort | 2006-2014 | 1-4 years | 137 | 11 | 8 |
|  | Australia | Tjokrowidjaja (2014) | PBCR | Cohort | 2001-2007 | 5-9 years | 6640 | 673 | 10 |
|  | New Zealand | James (2019) | PBCR | Cohort | 1993-2014 | 1-4 years | 1390 | 255 | 18 |

**
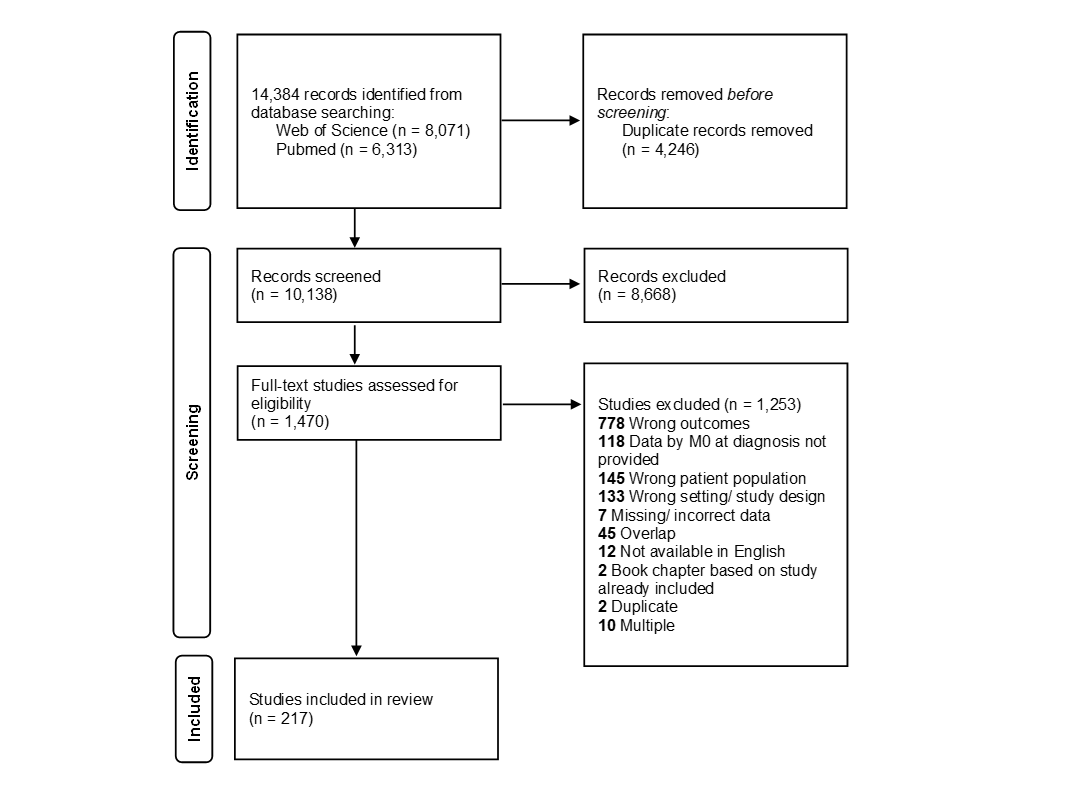
**

**eFigure 1:** PRISMA chart of study selection process

**eFigure 2:** Forest plot of the proportion of metastatic recurrences in women diagnosed with breast cancer at 1-4 years median follow-up in a) HR+ and b) HR- breast cancer patients and at 5-9 years median follow-up in c) HR+ and d) HR- breast cancer patients in studies that presented both subtypes.


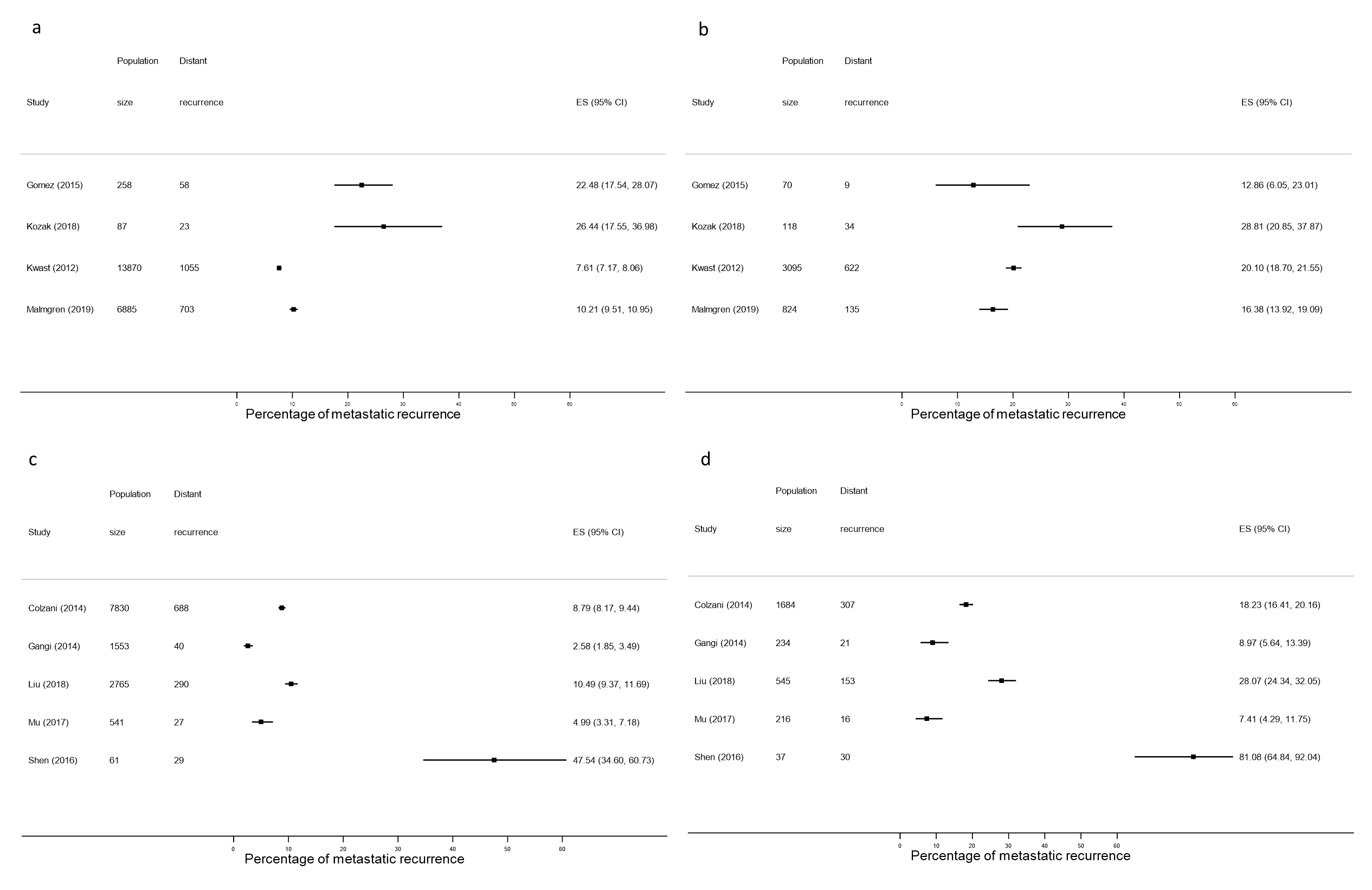


**eFigure 3:** Heatmap of results of risk of bias assessment of included studies

Risk of bias category

|  | **Risk of bias question** | | | | | | | | |  |
| --- | --- | --- | --- | --- | --- | --- | --- | --- | --- | --- |
| **Study author, year** | **1** | **2** | **3** | **4** | **5** | **6** | **7** | **8** | **9** | **Overall score** |
| Aalders, 2016[1] |  |  |  |  |  |  |  |  |  |  |
| Abdulwassi, 2020[2] |  |  |  |  |  |  |  |  |  |  |
| Acevedo, 2021[3] |  |  |  |  |  |  |  |  |  |  |
| Agarwal, 2020[4] |  |  |  |  |  |  |  |  |  |  |
| Ahn, 2017[5] |  |  |  |  |  |  |  |  |  |  |
| Al-Masri, 2021[6,7] |  |  |  |  |  |  |  |  |  |  |
| Alanko, 2021^7^ |  |  |  |  |  |  |  |  |  |  |
| Ali, 2022[8] |  |  |  |  |  |  |  |  |  |  |
| Anwar, 2020[9] |  |  |  |  |  |  |  |  |  |  |
| Atalay, 2015[10] |  |  |  |  |  |  |  |  |  |  |
| Barele, 2020[11] |  |  |  |  |  |  |  |  |  |  |
| Beecher, 2016[12] |  |  |  |  |  |  |  |  |  |  |
| Bhatti, 2014[13] |  |  |  |  |  |  |  |  |  |  |
| Bhatti, 2014 b[14] |  |  |  |  |  |  |  |  |  |  |
| Bjohle, 2019[15] |  |  |  |  |  |  |  |  |  |  |
| Bjorner, 2018[16] |  |  |  |  |  |  |  |  |  |  |
| Bodilsen, 2016[17] |  |  |  |  |  |  |  |  |  |  |
| Botteri, 2012[18] |  |  |  |  |  |  |  |  |  |  |
| Cabıoglu, 2021[19] |  |  |  |  |  |  |  |  |  |  |
| Canada, 2018[20] |  |  |  |  |  |  |  |  |  |  |
| Cancello, 2013[21] |  |  |  |  |  |  |  |  |  |  |
| Chan, 2012[22] |  |  |  |  |  |  |  |  |  |  |
| Chaudry, 2015[23] |  |  |  |  |  |  |  |  |  |  |
| Chen, 2015[24] |  |  |  |  |  |  |  |  |  |  |
| Cherng, 2020[25] |  |  |  |  |  |  |  |  |  |  |
| Chitapanarux, 2013[26] |  |  |  |  |  |  |  |  |  |  |
| Choi, 2017[27] |  |  |  |  |  |  |  |  |  |  |
| Cipolla, 2021[28] |  |  |  |  |  |  |  |  |  |  |
| Colzani, 2014[29] |  |  |  |  |  |  |  |  |  |  |
| Cortesi, 2013[30] |  |  |  |  |  |  |  |  |  |  |
| Crawford, 2013[31] |  |  |  |  |  |  |  |  |  |  |
| Crispo, 2013[32] |  |  |  |  |  |  |  |  |  |  |
| Cruz-Ku, 2020[33] |  |  |  |  |  |  |  |  |  |  |
| Cui, 2017[34] |  |  |  |  |  |  |  |  |  |  |
| Dackus, 2021[35] |  |  |  |  |  |  |  |  |  |  |
| Dackus, 2018[36] |  |  |  |  |  |  |  |  |  |  |
| Dawood, 2012[37] |  |  |  |  |  |  |  |  |  |  |
| Dieci, 2019[38] |  |  |  |  |  |  |  |  |  |  |
| Doepker, 2018[39] |  |  |  |  |  |  |  |  |  |  |
| Duraker, 2020[40] |  |  |  |  |  |  |  |  |  |  |
| Echavarria, 2017[41] |  |  |  |  |  |  |  |  |  |  |
| Elsayed, 2016[42] |  |  |  |  |  |  |  |  |  |  |
| Ess, 2018[43] |  |  |  |  |  |  |  |  |  |  |
| Eulenburg, 2016[44] |  |  |  |  |  |  |  |  |  |  |
| Fastner, 2016[45] |  |  |  |  |  |  |  |  |  |  |
| Fayanju, 2015[46] |  |  |  |  |  |  |  |  |  |  |
| Fehrenbacher, 2014[47] |  |  |  |  |  |  |  |  |  |  |
| Fontes-Sousa, 2020[48] |  |  |  |  |  |  |  |  |  |  |
| Fortunato, 2012[49] |  |  |  |  |  |  |  |  |  |  |
| Fredholm, 2016[50] |  |  |  |  |  |  |  |  |  |  |
| Gagliato Dde, 2014[51] |  |  |  |  |  |  |  |  |  |  |
| Gangi, 2014[52] |  |  |  |  |  |  |  |  |  |  |
| Garcia Fernandez, 2015 [53] |  |  |  |  |  |  |  |  |  |  |
| Ghavami, 2017[54] |  |  |  |  |  |  |  |  |  |  |
| Gobardhan, 2011[55] |  |  |  |  |  |  |  |  |  |  |
| Gomez, 2015[56] |  |  |  |  |  |  |  |  |  |  |
| Grassadonia, 2017[57] |  |  |  |  |  |  |  |  |  |  |
| Guiu, 2013[58] |  |  |  |  |  |  |  |  |  |  |
| Gunduz, 2015[59] |  |  |  |  |  |  |  |  |  |  |
| Han, 2015[60] |  |  |  |  |  |  |  |  |  |  |
| Harahap, 2017[61] |  |  |  |  |  |  |  |  |  |  |
| He, 2015[62] |  |  |  |  |  |  |  |  |  |  |
| Hirko, 2021[63] |  |  |  |  |  |  |  |  |  |  |
| Holzel, 2017[64] |  |  |  |  |  |  |  |  |  |  |
| Houvenaeghel, 2016[65] |  |  |  |  |  |  |  |  |  |  |
| Houze de l'Aulnoit, 2018[66] |  |  |  |  |  |  |  |  |  |  |
| Hu, 2020[67] |  |  |  |  |  |  |  |  |  |  |
| Imasato, 2010[68] |  |  |  |  |  |  |  |  |  |  |
| James, 2019[69] |  |  |  |  |  |  |  |  |  |  |
| Jamshed, 2015[70] |  |  |  |  |  |  |  |  |  |  |
| Jia, 2014[71] |  |  |  |  |  |  |  |  |  |  |
| Jo, 2015[72] |  |  |  |  |  |  |  |  |  |  |
| Joo, 2019[73] |  |  |  |  |  |  |  |  |  |  |
| Jueckstock, 2015[74] |  |  |  |  |  |  |  |  |  |  |
| Jung, 2019[75] |  |  |  |  |  |  |  |  |  |  |
| Jurrius, 2020[76] |  |  |  |  |  |  |  |  |  |  |
| Kang, 2019[77] |  |  |  |  |  |  |  |  |  |  |
| Kannan, 2022[78] |  |  |  |  |  |  |  |  |  |  |
| Kantelhardt, 2014[79] |  |  |  |  |  |  |  |  |  |  |
| Keilty, 2020[80] |  |  |  |  |  |  |  |  |  |  |
| Kim, 2011 [81] |  |  |  |  |  |  |  |  |  |  |
| Kim, 2016[82] |  |  |  |  |  |  |  |  |  |  |
| Kim, 2019[83] |  |  |  |  |  |  |  |  |  |  |
| Kiyoto, 2016[84] |  |  |  |  |  |  |  |  |  |  |
| Kozak, 2018[85] |  |  |  |  |  |  |  |  |  |  |
| Krenn-Pilko, 2015[86] |  |  |  |  |  |  |  |  |  |  |
| Kummel, 2015[87] |  |  |  |  |  |  |  |  |  |  |
| Kustic, 2019[88] |  |  |  |  |  |  |  |  |  |  |
| Kwast, 2012[89] |  |  |  |  |  |  |  |  |  |  |
| Kwon, 2022[90] |  |  |  |  |  |  |  |  |  |  |
| Laohavinij, 2013[91] |  |  |  |  |  |  |  |  |  |  |
| Larson, 2018[92] |  |  |  |  |  |  |  |  |  |  |
| Larson, 2018b[93] |  |  |  |  |  |  |  |  |  |  |
| Lee, 2015[94] |  |  |  |  |  |  |  |  |  |  |
| Lee, 2015b[95] |  |  |  |  |  |  |  |  |  |  |
| Lee, 2017[96] |  |  |  |  |  |  |  |  |  |  |
| Lee, 2018[97] |  |  |  |  |  |  |  |  |  |  |
| Li, 2021[98] |  |  |  |  |  |  |  |  |  |  |
| Libson, 2019[99] |  |  |  |  |  |  |  |  |  |  |
| Liikanen, 2018[100] |  |  |  |  |  |  |  |  |  |  |
| Liu, 2011[101] |  |  |  |  |  |  |  |  |  |  |
| Liu, 2016[102] |  |  |  |  |  |  |  |  |  |  |
| Liu, 2018[103] |  |  |  |  |  |  |  |  |  |  |
| Lu, 2013[104] |  |  |  |  |  |  |  |  |  |  |
| Luangdilok, 2014[105] |  |  |  |  |  |  |  |  |  |  |
| Luo, 2019[106] |  |  |  |  |  |  |  |  |  |  |
| Luo, 2022[107] |  |  |  |  |  |  |  |  |  |  |
| Ma, 2016[108] |  |  |  |  |  |  |  |  |  |  |
| Maanon, 2018[109] |  |  |  |  |  |  |  |  |  |  |
| Macfie, 2021[110] |  |  |  |  |  |  |  |  |  |  |
| Malmgren, 2019[111] |  |  |  |  |  |  |  |  |  |  |
| Marta, 2020[112] |  |  |  |  |  |  |  |  |  |  |
| Mazzarella, 2013[113,114] |  |  |  |  |  |  |  |  |  |  |
| Meattini, 2014[114,115] |  |  |  |  |  |  |  |  |  |  |
| Migdady, 2013 |  |  |  |  |  |  |  |  |  |  |
| Miller, 2017[116] |  |  |  |  |  |  |  |  |  |  |
| Minicozzi, 2013[117] |  |  |  |  |  |  |  |  |  |  |
| Miyake, 2015[118] |  |  |  |  |  |  |  |  |  |  |
| Miyashita, 2019[119] |  |  |  |  |  |  |  |  |  |  |
| Monrigal, 2011[120] |  |  |  |  |  |  |  |  |  |  |
| Morley, 2010[121] |  |  |  |  |  |  |  |  |  |  |
| Morris, 2012[122] |  |  |  |  |  |  |  |  |  |  |
| Mousavi, 2019[123] |  |  |  |  |  |  |  |  |  |  |
| Mu, 2017[124] |  |  |  |  |  |  |  |  |  |  |
| Mudduwa, 2018[125] |  |  |  |  |  |  |  |  |  |  |
| Musolino, 2011[126] |  |  |  |  |  |  |  |  |  |  |
| Nagar, 2015[127] |  |  |  |  |  |  |  |  |  |  |
| Naher, 2018[128] |  |  |  |  |  |  |  |  |  |  |
| Nedumpara, 2011[129] |  |  |  |  |  |  |  |  |  |  |
| Nogi 2021[130] |  |  |  |  |  |  |  |  |  |  |
| Orditura, 2016[131] |  |  |  |  |  |  |  |  |  |  |
| Orsaria, 2021[132] |  |  |  |  |  |  |  |  |  |  |
| Park, 2010[133] |  |  |  |  |  |  |  |  |  |  |
| Park, 2017[134] |  |  |  |  |  |  |  |  |  |  |
| Peng, 2011[135] |  |  |  |  |  |  |  |  |  |  |
| Perez, 2013[136] |  |  |  |  |  |  |  |  |  |  |
| Pomponio, 2020[137] |  |  |  |  |  |  |  |  |  |  |
| Prajoko, 2019[138] |  |  |  |  |  |  |  |  |  |  |
| Purushotham, 2014[139] |  |  |  |  |  |  |  |  |  |  |
| Qiu, 2016[140] |  |  |  |  |  |  |  |  |  |  |
| Quan, 2017[141] |  |  |  |  |  |  |  |  |  |  |
| Ragage, 2010[142] |  |  |  |  |  |  |  |  |  |  |
| Raghavan, 2019[143] |  |  |  |  |  |  |  |  |  |  |
| Rahal, 2015[144] |  |  |  |  |  |  |  |  |  |  |
| Rakha, 2012[145] |  |  |  |  |  |  |  |  |  |  |
| Raouf, 2020[146] |  |  |  |  |  |  |  |  |  |  |
| Ratosa, 2021[147] |  |  |  |  |  |  |  |  |  |  |
| Rosendahl, 2015[148] |  |  |  |  |  |  |  |  |  |  |
| Rossi, 2015[149] |  |  |  |  |  |  |  |  |  |  |
| Ryu, 2018[150] |  |  |  |  |  |  |  |  |  |  |
| Sabiani, 2016[151] |  |  |  |  |  |  |  |  |  |  |
| Saha, 2018[152] |  |  |  |  |  |  |  |  |  |  |
| Saini, 2018[153] |  |  |  |  |  |  |  |  |  |  |
| Salem, 2017[154] |  |  |  |  |  |  |  |  |  |  |
| Sanchez, 2010[155] |  |  |  |  |  |  |  |  |  |  |
| Sanpaolo, 2011[156] |  |  |  |  |  |  |  |  |  |  |
| Sanpaolo, 2012[157] |  |  |  |  |  |  |  |  |  |  |
| Sato, 2020[158] |  |  |  |  |  |  |  |  |  |  |
| Saw, 2019[159] |  |  |  |  |  |  |  |  |  |  |
| Schaffar, 2019[160] |  |  |  |  |  |  |  |  |  |  |
| Schmidt, 2011[161] |  |  |  |  |  |  |  |  |  |  |
| Schmidt, 2016[162] |  |  |  |  |  |  |  |  |  |  |
| Shandiz, 2016[163] |  |  |  |  |  |  |  |  |  |  |
| Sharma, 2018[164] |  |  |  |  |  |  |  |  |  |  |
| Sharp, 2021[165] |  |  |  |  |  |  |  |  |  |  |
| Shen, 2016[166] |  |  |  |  |  |  |  |  |  |  |
| Shiao, 2017[167] |  |  |  |  |  |  |  |  |  |  |
| Shim, 2018[168] |  |  |  |  |  |  |  |  |  |  |
| Shohdy, 2021[169] |  |  |  |  |  |  |  |  |  |  |
| Simons, 2021[170] |  |  |  |  |  |  |  |  |  |  |
| Smith, 2017[171] |  |  |  |  |  |  |  |  |  |  |
| Song, 2011[172] |  |  |  |  |  |  |  |  |  |  |
| Sopik, 2019[173] |  |  |  |  |  |  |  |  |  |  |
| Soran, 2019[174] |  |  |  |  |  |  |  |  |  |  |
| Spring, 2017[175] |  |  |  |  |  |  |  |  |  |  |
| Sun, 2021[176] |  |  |  |  |  |  |  |  |  |  |
| Tjokrowidjaja, 2014[177] |  |  |  |  |  |  |  |  |  |  |
| Tokuda, 2017[178] |  |  |  |  |  |  |  |  |  |  |
| Tong, 2020[179] |  |  |  |  |  |  |  |  |  |  |
| Tonyali, 2013[180] |  |  |  |  |  |  |  |  |  |  |
| Tovanabutra, 2020[181] |  |  |  |  |  |  |  |  |  |  |
| Tung, 2014[182] |  |  |  |  |  |  |  |  |  |  |
| Tzikas, 2020[183] |  |  |  |  |  |  |  |  |  |  |
| Ulas, 2015[184] |  |  |  |  |  |  |  |  |  |  |
| Van Asten, 2019[185] |  |  |  |  |  |  |  |  |  |  |
| Vu Hong, 2019[186] |  |  |  |  |  |  |  |  |  |  |
| Wang, 2017[187] |  |  |  |  |  |  |  |  |  |  |
| Wang, 2019[188] |  |  |  |  |  |  |  |  |  |  |
| Wang, 2020[189] |  |  |  |  |  |  |  |  |  |  |
| Wang, 2021[190] |  |  |  |  |  |  |  |  |  |  |
| Wen, 2017[191] |  |  |  |  |  |  |  |  |  |  |
| White, 2016[192] |  |  |  |  |  |  |  |  |  |  |
| Wirtz, 2013[193] |  |  |  |  |  |  |  |  |  |  |
| Wobb, 2016[194] |  |  |  |  |  |  |  |  |  |  |
| Wu, 2014[195] |  |  |  |  |  |  |  |  |  |  |
| Wu, 2014[196] |  |  |  |  |  |  |  |  |  |  |
| Wu, 2016[197] |  |  |  |  |  |  |  |  |  |  |
| Xu, 2022[198] |  |  |  |  |  |  |  |  |  |  |
| Xue, 2015[199] |  |  |  |  |  |  |  |  |  |  |
| Yamada, 2018[200] |  |  |  |  |  |  |  |  |  |  |
| Yamada, 2020[201] |  |  |  |  |  |  |  |  |  |  |
| Yang, 2018[202] |  |  |  |  |  |  |  |  |  |  |
| Ye, 2020[203] |  |  |  |  |  |  |  |  |  |  |
| Yoon, 2019[204] |  |  |  |  |  |  |  |  |  |  |
| Zaky, 2011[205] |  |  |  |  |  |  |  |  |  |  |
| Zemni, 2017[206] |  |  |  |  |  |  |  |  |  |  |
| Zhang, 2014[207] |  |  |  |  |  |  |  |  |  |  |
| Zhang, 2018[208] |  |  |  |  |  |  |  |  |  |  |
| Zhang, 2021[209] |  |  |  |  |  |  |  |  |  |  |
| Zhao, 2015[210] |  |  |  |  |  |  |  |  |  |  |
| Zhong, 2019[211] |  |  |  |  |  |  |  |  |  |  |
| Zouzoulas, 2020[212] |  |  |  |  |  |  |  |  |  |  |
| Zumsteg, 2013[213] |  |  |  |  |  |  |  |  |  |  |
| van Steenhoven, 2020[214] |  |  |  |  |  |  |  |  |  |  |
| van Laar, 2013[215] |  |  |  |  |  |  |  |  |  |  |
| vanRoozendaal, 2016[216] |  |  |  |  |  |  |  |  |  |  |
| van den Hurk, 2011[217] |  |  |  |  |  |  |  |  |  |  |

^*Questions for risk of bias assessment: 1. Was the study population a close representation of the target population in relation to relevant variables? 2. Was the sampling frame a true or close representation of the target population? 3. Was some form of random selection used to select the sample, or was a population-based study undertaken? 4. Was the likelihood of missing information on distant recurrence and completeness of follow-up minimal? 5. Were data collected directly from patient examination, record linkage or scrutinising medical records? 6. Was an acceptable definition of distant recurrence used in the study? 7. Was the study instrument that measured the parameter of interest (e.g. proportion of metastatic distant recurrence) shown to be reliable and valid? 8. Was the same mode of data collection used for all subjects? 9. Were the numerator(s) and denominator(s) for the parameter of interest appropriate?^

**References**

1 Aalders KC, Postma EL, Strobbe LJ, *et al.* Contemporary Locoregional Recurrence Rates in Young Patients With Early-Stage Breast Cancer. *Journal of Clinical Oncology*. 2016;34:2107-+.

2 Abdulwassi HK, Amer IT, Alhibshi AH, *et al.* Recurrence rates and long-term survival factors in young women with breast cancer. *Saudi Med J*. 2020;41:393–9.

3 Acevedo F, Petric M, Walbaum B, *et al.* Better overall survival in patients who achieve pathological complete response after neoadjuvant chemotherapy for breast cancer in a Chilean public hospital. *Ecancermedicalscience*. 2021;15:1185.

4 Agarwal R, Unnikrishnan UG, Keechilat P, *et al.* Pathological Complete Response in Locally Advanced Breast Cancer after Neoadjuvant Chemotherapy: Survival Outcome and Its Relevance as a Surrogate End Point. *South Asian J Cancer*. 2020;9:136–40.

5 Ahn KJ, Park J, Choi Y. Lymphovascular invasion as a negative prognostic factor for triple-negative breast cancer after surgery. *Radiat Oncol J*. 2017;35:332–9.

6 Al-Masri M, Aljalabneh B, Al-Najjar H, *et al.* Effect of time to breast cancer surgery after neoadjuvant chemotherapy on survival outcomes. *Breast Cancer Res Treat*. 2021;186:7–13.

7 Alanko J, Tanner M, Vanninen R, *et al.* Triple-negative and HER2-positive breast cancers found by mammography screening show excellent prognosis. *Breast Cancer Res Treat*. 2021;187:267–74.

8 Ali S, Hendry J, Le D, *et al.* Efficacy of adjuvant trastuzumab in women with HER2-positive T1a or bN0M0 breast cancer: a population-based cohort study. *Sci Rep*. 2022;12:1068–1068.

9 Anwar SL, Avanti WS, Nugroho AC, *et al.* Risk factors of distant metastasis after surgery among different breast cancer subtypes: a hospital-based study in Indonesia. *World J Surg Oncol*. 2020;18:117.

10 Atalay C, Kucuk AI. The impact of weight gain during adjuvant chemotherapy on survival in breast cancer. *Turk J Surg*. 2015;31:124–7.

11 van Barele M, Heemskerk-Gerritsen BAM, van Doorn HC, *et al.* The impact of menstruation persistence or recovery after chemotherapy on survival in young patients with hormone receptor negative breast cancer. *Breast*. 2020;52:102–9.

12 Beecher SM, O’Leary DP, McLaughlin R, *et al.* Influence of complications following immediate breast reconstruction on breast cancer recurrence rates. *British Journal of Surgery*. 2016;103:391–8.

13 Bhatti AB, Jamshed A, Khan A, *et al.* Comparison between early and late onset breast cancer in Pakistani women undergoing breast conservative therapy: is there any difference? *Asian Pac J Cancer Prev*. 2014;15:5331–6.

14 Bhatti AB, Khan AI, Siddiqui N, *et al.* Outcomes of triple-negative versus non-triple-negative breast cancers managed with breast-conserving therapy. *Asian Pac J Cancer Prev*. 2014;15:2577–81.

15 Bjohle J, Onjukka E, Rintela N, *et al.* Post-mastectomy radiation therapy with or without implant-based reconstruction is safe in terms of clinical target volume coverage and survival - A matched cohort study. *Radiotherapy and Oncology*. 2019;131:229–36.

16 Bjorner S, Rosendahl AH, Tryggvadottir H, *et al.* Coffee Is Associated With Lower Breast Tumor Insulin-Like Growth Factor Receptor 1 Levels in Normal-Weight Patients and Improved Prognosis Following Tamoxifen or Radiotherapy Treatment. *Front Endocrinol (Lausanne)*. 2018;9.

17 Bodilsen A, Offersen B V, Christiansen P, *et al.* Pattern of relapse after breast conserving therapy, a study of 1519 early breast cancer patients treated in the Central Region of Denmark 2000-2009. *Acta Oncol (Madr)*. 2016;55:964–9.

18 Botteri E, Gentilini O, Rotmensz N, *et al.* Mastectomy without radiotherapy: outcome analysis after 10 years of follow-up in a single institution. *Breast Cancer Res Treat*. 2012;134:1221–8.

19 Cabioglu N, Karanlik H, Yildirim N, *et al.* Favorable outcome with sentinel lymph node biopsy alone after neoadjuvant chemotherapy in clinically node positive breast cancer at diagnosis: Turkish Multicentric NEOSENTI-TURK MF-18-02-study. *EJSO*. 2021;47:2506–14.

20 Baena Canada JM, Gamez Casado S, Rodriguez Perez L, *et al.* Evaluation of non-genomic, clinical risk and survival results in endocrine-sensitive, HER-2 negative, lymph node negative breast cancer. *Med Clin (Barc)*. 2018;151:469–75.

21 Cancello G, Maisonneuve P, Mazza M, *et al.* Pathological features and survival outcomes of very young patients with early breast cancer: How much is ‘very young’? *Breast*. 2013;22:1046–51.

22 Chan A, Willsher PC, Hastrich DJ, *et al.* Preoperative taxane-based chemotherapy in a standardized protocol for locally advanced breast cancer. *Asia Pac J Clin Oncol*. 2012;8:62–70.

23 Chaudry M, Lei X, Gonzalez-Angulo AM, *et al.* Recurrence and survival among breast cancer patients achieving a pathological complete response to neoadjuvant chemotherapy. *Breast Cancer Res Treat*. 2015;153:417–23.

24 Chen ZH, Xu Y, Shu JD, *et al.* Breast-conserving surgery versus modified radical mastectomy in treatment of early stage breast cancer: A retrospective study of 107 cases. *J Cancer Res Ther*. 2015;11:C29–31.

25 Cherng HR, Rice SR, Hamza M, *et al.* Patterns of Failure in Triple Negative Breast Cancer Patients in an Urban, Predominately Black Population. *J Racial Ethn Health Disparities*. 2021;8:1035–46.

26 Chitapanarux I, Trakultivakorn H, Srisukho S, *et al.* Real-world outcomes of different treatments in the management of patients with HER-2 positive breast cancer: a retrospective study. *J Med Assoc Thai*. 2013;96:709–15.

27 Choi M, Park YH, Ahn JS, *et al.* Evaluation of Pathologic Complete Response in Breast Cancer Patients Treated with Neoadjuvant Chemotherapy: Experience in a Single Institution over a 10-Year Period. *J Pathol Transl Med*. 2017;51:69–78.

28 Cipolla C, Galvano A, Vieni S, *et al.* Effects of the number of removed lymph nodes on survival outcome in patients with sentinel node-negative breast cancer. *World J Surg Oncol*. 2021;19:306.

29 Colzani E, Johansson AL V, Liljegren A, *et al.* Time-dependent risk of developing distant metastasis in breast cancer patients according to treatment, age and tumour characteristics. *Br J Cancer*. 2014;110:1378–84.

30 Cortesi L, Marcheselli L, Guarneri V, *et al.* Tumor size, node status, grading, HER2 and estrogen receptor status still retain a strong value in patients with operable breast cancer diagnosed in recent years. *Int J Cancer*. 2013;132:E58–65.

31 Crawford JD, Ansteth M, Barnett J, *et al.* Routine completion axillary lymph node dissection for positive sentinel nodes in patients undergoing mastectomy is not associated with improved local control. *Am J Surg*. 2013;205:581–4.

32 Crispo A, Barba M, D’Aiuto G, *et al.* Molecular profiles of screen detected vs. symptomatic breast cancer and their impact on survival: results from a clinical series. *BMC Cancer*. 2013;13.

33 De la Cruz-Ku GA, Chambergo-Michilot D, Valcarcel B, *et al.* Lymph node ratio as best prognostic factor in triple-negative breast cancer patients with residual disease after neoadjuvant chemotherapy. *Breast J*. 2020;26:1659–66.

34 Cui JX. Comparative study on the effect of breast conserving surgery and modified radical operation in patients with early stage breast cancer. *Biomedical Research-India*. 2017;28:5930–3.

35 Dackus G, Jóźwiak K, Sonke GS, *et al.* Adjuvant Aromatase Inhibitors or Tamoxifen Following Chemotherapy for Perimenopausal Breast Cancer Patients. *J Natl Cancer Inst*. 2021;113:1506–14.

36 Dackus GMHE, Jóźwiak K, Sonke GS, *et al.* Optimal adjuvant endocrine treatment of ER+/HER2+ breast cancer patients by age at diagnosis: A population-based cohort study. *Eur J Cancer*. 2018;90:92–101.

37 Dawood S, Lei X, Litton JK, *et al.* Impact of body mass index on survival outcome among women with early stage triple-negative breast cancer. *Clin Breast Cancer*. 2012;12:364–72.

38 Dieci M V, Tsvetkova V, Griguolo G, *et al.* Androgen Receptor Expression and Association With Distant Disease-Free Survival in Triple Negative Breast Cancer: Analysis o 263 Patients Treated With Standard Therapy for Stage I-III Disease. *Front Oncol*. 2019;9.

39 Doepker MP, Holt SD, Durkin MW, *et al.* Triple-Negative Breast Cancer: A Comparison of Race and Survival. *American Surgeon*. 2018;84:881–8.

40 Duraker N, Hot S, Akan A, *et al.* A Comparison of the Clinicopathological Features, Metastasis Sites and Survival Outcomes of Invasive Lobular, Invasive Ductal and Mixed Invasive Ductal and Lobular Breast Carcinoma. *Eur J Breast Health*. 2020;16:22–31.

41 Echavarria I, Granja M, Bueno C, *et al.* Multicenter analysis of neoadjuvant docetaxel, carboplatin, and trastuzumab in HER2-positive breast cancer. *Breast Cancer Res Treat*. 2017;162:181–9.

42 Elsayed M, Alhussini M, Basha A, *et al.* Analysis of loco-regional and distant recurrences in breast cancer after conservative surgery. *World J Surg Oncol*. 2016;14.

43 Ess SM, Herrmann C, Bouchardy C, *et al.* Impact of subtypes and comorbidities on breast cancer relapse and survival in population-based studies. *Breast*. 2018;41:151–8.

44 Eulenburg C, Schroeder J, Obi N, *et al.* A Comprehensive Multistate Model Analyzing Associations of Various Risk Factors With the Course of Breast Cancer in a Population-Based Cohort of Breast Cancer Cases. *Am J Epidemiol*. 2016;183:325–34.

45 Fastner G, Hauser-Kronberger C, Moder A, *et al.* Survival and local control rates of triple-negative breast cancer patients treated with boost-IOERT during breast-conserving surgery. *Strahlentherapie Und Onkologie*. 2016;192:1–7.

46 Fayanju OM, Nwaogu I, Jeffe DB, *et al.* Pathological complete response in breast cancer patients following neoadjuvant chemotherapy at a Comprehensive Cancer Center: The natural history of an elusive prognosticator. *Mol Clin Oncol*. 2015;3:775–80.

47 Fehrenbacher L, Capra AM, Quesenberry Jr. CP, *et al.* Distant invasive breast cancer recurrence risk in human epidermal growth factor receptor 2-positive T1a and T1b node-negative localized breast cancer diagnosed from 2000 to 2006: a cohort from an integrated health care delivery system. *J Clin Oncol*. 2014;32:2151–8.

48 Fontes-Sousa M, Lobo J, Lobo S, *et al.* Digital imaging-assisted quantification of H3K27me3 immunoexpression in luminal A/B-like, HER2-negative, invasive breast cancer predicts patient survival and risk of recurrence. *Molecular Medicine*. 2020;26.

49 Fortunato L, Mascaro A, Poccia I, *et al.* Lobular breast cancer: same survival and local control compared with ductal cancer, but should both be treated the same way? analysis of an institutional database over a 10-year period. *Ann Surg Oncol*. 2012;19:1107–14.

50 Fredholm H, Magnusson K, Lindström LS, *et al.* Long-term outcome in young women with breast cancer: a population-based study. *Breast Cancer Res Treat*. 2016;160:131–43.

51 Gagliato Dde M, Gonzalez-Angulo AM, Lei X, *et al.* Clinical impact of delaying initiation of adjuvant chemotherapy in patients with breast cancer. *J Clin Oncol*. 2014;32:735–44.

52 Gangi A, Chung A, Mirocha J, *et al.* Breast-Conserving Therapy for Triple-Negative Breast Cancer. *JAMA Surg*. 2014;149:252–8.

53 Fernandez AG, Chabrera C, Font MG, *et al.* Differential patterns of recurrence and specific survival between luminal A and luminal B breast cancer according to recent changes in the 2013 St Gallen immunohistochemical classification. *Clinical & Translational Oncology*. 2015;17:238–46.

54 Ghavami V, Mahmoudi M, Rahimi Foroushani A, *et al.* Long-Term Disease-Free Survival of Non-Metastatic Breast Cancer Patients in Iran: A Survival Model with Competing Risks Taking Cure Fraction and Frailty into Account. *Asian Pac J Cancer Prev*. 2017;18:2825–32.

55 Gobardhan PD, Elias SG, Madsen EVE, *et al.* Prognostic Value of Lymph Node Micrometastases in Breast Cancer: A Multicenter Cohort Study. *Ann Surg Oncol*. 2011;18:1657–64.

56 Gomez R, Ossa CA, Montoya ME, *et al.* Impact of immunohistochemistry-based molecular subtype on chemosensitivity and survival in Hispanic breast cancer patients following neoadjuvant chemotherapy. *Ecancermedicalscience*. 2015;9:562.

57 Grassadonia A, Vici P, Gamucci T, *et al.* Long-term outcome of breast cancer patients with pathologic N3a lymph node stage. *Breast*. 2017;32:79–86.

58 Guiu S, Arnould L, Bonnetain F, *et al.* Pathological response and survival after neoadjuvant therapy for breast cancer: a 30-year study. *Breast*. 2013;22:301–8.

59 Gunduz S, Goksu SS, Arslan D, *et al.* Factors affecting disease-free survival in patients with human epidermal growth factor receptor 2-positive breast cancer who receive adjuvant trastuzumab. *Mol Clin Oncol*. 2015;3:1109–12.

60 Han Y, Li Q, Xu BH, *et al.* Adjuvant chemotherapy may improve survival of patients with luminal A breast cancer and positive lymph nodes. *Genetics and Molecular Research*. 2015;14:8563–73.

61 Arif Harahap W, Ramadhan, Khambri D, *et al.* Outcomes of Trastuzumab Therapy for 6 and 12 Months in Indonesian National Health Insurance System Clients with Operable HER2-Positive Breast Cancer. *Asian Pac J Cancer Prev*. 2017;18:1151–6.

62 He ZY, Wu SG, Zhou J, *et al.* Postmastectomy Radiotherapy Improves Disease-Free Survival of High Risk of Locoregional Recurrence Breast Cancer Patients with T1-2 and 1 to 3 Positive Nodes. *PLoS One*. 2015;10.

63 Hirko KA, Regan MM, Remolano MC, *et al.* Dermal Lymphatic Invasion, Survival, and Time to Recurrence or Progression in Inflammatory Breast Cancer. *Am J Clin Oncol*. 2021;44:449–55.

64 Holzel D, Eckel R, Bauerfeind I, *et al.* Improved systemic treatment for early breast cancer improves cure rates, modifies metastatic pattern and shortens post-metastatic survival: 35-year results from the Munich Cancer Registry. *J Cancer Res Clin Oncol*. 2017;143:1701–12.

65 Houvenaeghel G, Sabatier R, Reyal F, *et al.* Axillary lymph node micrometastases decrease triple-negative early breast cancer survival. *Br J Cancer*. 2016;115:1024–31.

66 Houze de l’Aulnoit A, Rogoz B, Pincon C, *et al.* Metastasis-free interval in breast cancer patients: Thirty-year trends and time dependency of prognostic factors. A retrospective analysis based on a single institution experience. *Breast*. 2018;37:80–8.

67 Hu Y, Wang S, Ding N, *et al.* Platelet/Lymphocyte Ratio Is Superior to Neutrophil/Lymphocyte Ratio as a Predictor of Chemotherapy Response and Disease-free Survival in Luminal B-like (HER2(-)) Breast Cancer. *Clin Breast Cancer*. 2020.

68 Imasato M, Shimazu K, Tamaki Y, *et al.* Long-term follow-up results of breast cancer patients with sentinel lymph node biopsy using periareolar injection. *Am J Surg*. 2010;199:442–6.

69 James M, Dixit A, Robinson B, *et al.* Outcomes for Patients with Non-metastatic Triple-negative Breast Cancer in New Zealand. *Clin Oncol*. 2019;31:17–24.

70 Jamshed A, Shah MA, Syed AA, *et al.* Clinical outcome of primary non-metastatic breast cancer: A single institution experience. *Indian J Cancer*. 2015;52:119-+.

71 Jia WJ, Jia HX, Feng HY, *et al.* HER2-enriched Tumors Have the Highest Risk of Local Recurrence in Chinese Patients Treated with Breast Conservation Therapy. *Asian Pacific Journal of Cancer Prevention*. 2014;15:315–20.

72 Jo JE, Kim JY, Lee SH, *et al.* Preoperative 18F-FDG PET/CT predicts disease-free survival in patients with primary invasive ductal breast cancer. *Acta radiol*. 2015;56:1463–70.

73 Joo JH, Kim SS, Son BH, *et al.* Axillary Lymph Node Dissection Does Not Improve Post-mastectomy Overall or Disease-Free Survival among Breast Cancer Patients with 1-3 Positive Nodes. *Cancer Res Treat*. 2019;51:1011–21.

74 Jueckstock J, Kasch F, Jaeger B, *et al.* Adjuvant therapeutic decisions in elderly breast cancer patients: the role of chemotherapy in a retrospective analysis. *Arch Gynecol Obstet*. 2015;292:1101–7.

75 Jung SU, Sohn G, Kim J, *et al.* Survival outcome of adjuvant endocrine therapy alone for patients with lymph node-positive, hormone-responsive, HER2-negative breast cancer. *Asian J Surg*. 2019;42:914–21.

76 Jurrius P, Green T, Garmo H, *et al.* Invasive breast cancer over four decades reveals persisting poor metastatic outcomes in treatment resistant subgroup - the ‘ATRESS’ phenomenon. *Breast*. 2020;50:39–48.

77 Kang YJ, Lee HB, Kim YG, *et al.* Ki-67 Expression is a Significant Prognostic Factor Only When Progesterone Receptor Expression is Low in Estrogen Receptor-Positive and HER2-Negative Early Breast Cancer. *J Oncol*. 2019;2019.

78 Kannan J, Mohanty S, Saklani A, *et al.* Pattern of Recurrence and Survival Outcomes in Non-Metastatic Triple-Negative Breast Cancer; A Retrospective Analysis. *TURK ONKOLOJI DERGISI-TURKISH JOURNAL OF ONCOLOGY*.

79 Kantelhardt EJ, Zerche P, Mathewos A, *et al.* Breast cancer survival in Ethiopia: A cohort study of 1,070 women. *Int J Cancer*. 2014;135:702–9.

80 Keilty D, Namini SN, Swain M, *et al.* Patterns of Recurrence and Predictors of Survival in Breast Cancer Patients Treated with Neoadjuvant Chemotherapy, Surgery, and Radiation. *Int J Radiat Oncol Biol Phys*. 2020;108:676–85.

81 Kim K, Chie EK, Han W, *et al.* Age <40Years is an independent prognostic factor predicting inferior overall survival in patients treated with breast conservative therapy. *Breast J*. 2011;17:75–8.

82 Kim TH, Yoon JK, Kang DK, *et al.* Value of volume-based metabolic parameters for predicting survival in breast cancer patients treated with neoadjuvant chemotherapy. *Medicine*. 2016;95.

83 Kim JY, Kim JJ, Hwangbo L, *et al.* Diffusion-weighted Imaging of Invasive Breast Cancer: Relationship to Distant Metastasis-free Survival. *Radiology*. 2019;291:299–306.

84 Kiyoto S, Sugawara Y, Hosokawa K, *et al.* Predictive Ability of (18)F-fluorodeoxyglucose Positron Emission Tomography/computed Tomography for Pathological Complete Response and Prognosis after Neoadjuvant Chemotherapy in Triple-negative Breast Cancer Patients. *Asia Ocean J Nucl Med Biol*. 2016;4:3–11.

85 Kozak MM, Jacobson CE, von Eyben R, *et al.* Patterns of Distant Failure by Intrinsic Breast Cancer Subtype in Premenopausal Women Treated With Neoadjuvant Chemotherapy. *Clin Breast Cancer*. 2018;18:E1077–85.

86 Krenn-Pilko S, Langsenlehner U, Stojakovic T, *et al.* An elevated preoperative plasma fibrinogen level is associated with poor disease-specific and overall survival in breast cancer patients. *Breast*. 2015;24:667–72.

87 Kummel A, Kummel S, Barinoff J, *et al.* Prognostic Factors for Local, Loco-regional and Systemic Recurrence in Early-stage Breast Cancer. *Geburtshilfe Frauenheilkd*. 2015;75:710–8.

88 Kustic D, Lovasic F, Belac-Lovasic I, *et al.* Impact of HER2 receptor status on axillary nodal burden in patients with non-luminal A invasive ductal breast carcinoma. *Rev Med Chil*. 2019;147:557–67.

89 Kwast AB, Groothuis-Oudshoorn KC, Grandjean I, *et al.* Histological type is not an independent prognostic factor for the risk pattern of breast cancer recurrences. *Breast Cancer Res Treat*. 2012;135:271–80.

90 Kwon BR, Shin SU, Kim SY, *et al.* Microcalcifications and Peritumoral Edema Predict Survival Outcome in Luminal Breast Cancer Treated with Neoadjuvant Chemotherapy. *Radiology*. 2022;211509.

91 Laohavinij S, Ruikchuchit K, Maneechavakajorn J. Survival and prognostic factors of stage I-III breast cancer. *J Med Assoc Thai*. 2013;96 Suppl 3:S23-34.

92 Larson KE, Grobmyer SR, Valente SA. Evaluation of recurrence patterns and survival in modern series of young women with breast cancer. *Breast Journal*. 2018;24:749–54.

93 Larson KE, Valente SA, Shah C, *et al.* Oncotype testing in patients undergoing intraoperative radiation for breast cancer. *Mol Clin Oncol*. 2018;9:535–8.

94 Lee JY, Lim SH, Lee MY, *et al.* Impact on Survival of Regular Postoperative Surveillance for Patients with Early Breast Cancer. *Cancer Res Treat*. 2015;47:765–73.

95 Lee Y, Kang E, Lee AS, *et al.* Outcomes and recurrence patterns according to breast cancer subtypes in Korean women. *Breast Cancer Res Treat*. 2015;151:183–90.

96 Lee SJ, Kang BW, Kim JG, *et al.* AQP5 Variants Affect Tumoral Expression of AQP5 and Survival in Patients with Early Breast Cancer. *Oncology*. 2017;92:153–60.

97 Lee SB, Sohn G, Kim J, *et al.* Survival analysis according to period and analysis of the factors influencing changes in survival in patients with recurrent breast cancer: a large-scale, single-center study. *Breast Cancer*. 2018;25:639–49.

98 Li Y, Lu S, Zhang Y, *et al.* Loco-regional recurrence trend and prognosis in young women with breast cancer according to molecular subtypes: analysis of 1099 cases. *World J Surg Oncol*. 2021;19:113.

99 Libson S, Perez E, Takita C, *et al.* Post Mastectomy Radiation For Stage II Breast Cancer Patients with T1/T2 Lesions. *Eur J Breast Health*. 2019;15:71–5.

100 Liikanen JS, Leidenius MH, Joensuu H, *et al.* Prognostic value of isolated tumour cells in sentinel lymph nodes in early-stage breast cancer: a prospective study. *Br J Cancer*. 2018;118:1529–35.

101 Liu FF, Lang RG, Zhao J, *et al.* CD8(+) cytotoxic T cell and FOXP3(+) regulatory T cell infiltration in relation to breast cancer survival and molecular subtypes. *Breast Cancer Res Treat*. 2011;130:645–55.

102 Liu YL, Saraf A, Lee SM, *et al.* Lymphovascular invasion is an independent predictor of survival in breast cancer after neoadjuvant chemotherapy. *Breast Cancer Res Treat*. 2016;157:555–64.

103 Liu Z, Sahli Z, Wang Y, *et al.* Young age at diagnosis is associated with worse prognosis in the Luminal A breast cancer subtype: a retrospective institutional cohort study. *Breast Cancer Res Treat*. 2018;172:689–702.

104 Lu C, Xu H, Chen X, *et al.* Irradiation after surgery for breast cancer patients with primary tumours and one to three positive axillary lymph nodes: yes or no? *Current Oncology*. 2013;20:E585–92.

105 Luangdilok S, Samarnthai N, Korphaisarn K. Association between Pathological Complete Response and Outcome Following Neoadjuvant Chemotherapy in Locally Advanced Breast Cancer Patients. *J Breast Cancer*. 2014;17:376–85.

106 Luo J, Jin K, Chen X, *et al.* Internal Mammary Node Irradiation (IMNI) Improves Survival Outcome for Patients With Clinical Stage II-III Breast Cancer After Preoperative Systemic Therapy. *Int J Radiat Oncol Biol Phys*. 2019;103:895–904.

107 Luo C, Zhong X, Fan Y, *et al.* Clinical characteristics and survival outcome of patients with estrogen receptor low positive breast cancer. *Breast*. 2022;63:24–8.

108 Ma RM, Chen CZ, Zhang W, *et al.* Prognostic Value of Chemotherapy-Induced Neutropenia at the First Cycle in Invasive Breast Cancer. *Medicine*. 2016;95.

109 Maanon J, Perez D, Rhode A, *et al.* High serum vascular endothelial growth factor C predicts better relapse-free survival in early clinically node-negative breast cancer. *Oncotarget*. 2018;9:28131–40.

110 Macfie R, Aks C, Panwala K, *et al.* Breast conservation therapy confers survival and distant recurrence advantage over mastectomy for stage II Triple Negative Breast cancer. *Am J Surg*. 2021;221:809–12.

111 Malmgren J, Hurlbert M, Atwood M, *et al.* Examination of a paradox: recurrent metastatic breast cancer incidence decline without improved distant disease survival: 1990-2011. *Breast Cancer Res Treat*. 2019;174:505–14.

112 Marta GN, AlBeesh R, Pereira AAL, *et al.* The impact on clinical outcomes of post-operative radiation therapy delay after neoadjuvant chemotherapy in patients with breast cancer: A multicentric international study. *Breast*. 2020;54:46–51.

113 Mazzarella L, Disalvatore D, Bagnardi V, *et al.* Obesity increases the incidence of distant metastases in oestrogen receptor-negative human epidermal growth factor receptor 2-positive breast cancer patients. *Eur J Cancer*. 2013;49:3588–97.

114 Meattini I, Cecchini S, Muntoni C, *et al.* Cutaneous and cardiac toxicity of concurrent trastuzumab and adjuvant breast radiotherapy: a single institution series. *Med Oncol*. 2014;31:891.

115 Migdady Y, Sakr BJ, Sikov WM, *et al.* Adjuvant chemotherapy in T1a/bN0 HER2-positive or triple-negative breast cancers: Application and outcomes. *Breast*. 2013;22:793–8.

116 Miller DH, Pflederer N, Malouff T, *et al.* Analysis of treatment effectiveness and complications associated with MammoSite (TM) breast brachytherapy in patients treated at a single institution. *J Radiat Oncol*. 2017;6:353–60.

117 Minicozzi P, Bella F, Toss A, *et al.* Relative and disease-free survival for breast cancer in relation to subtype: a population-based study. *J Cancer Res Clin Oncol*. 2013;139:1569–77.

118 Miyake T, Ito T, Yanai A, *et al.* C4.4A highly expressed in HER2-positive human breast cancers may indicate a good prognosis. *Breast Cancer*. 2015;22:366–73.

119 Miyashita M, Niikura N, Kumamaru H, *et al.* Role of Postmastectomy Radiotherapy After Neoadjuvant Chemotherapy in Breast Cancer Patients: A Study from the Japanese Breast Cancer Registry. *Ann Surg Oncol*. 2019;26:2475–85.

120 Monrigal E, Dauplat J, Gimbergues P, *et al.* Mastectomy with immediate breast reconstruction after neoadjuvant chemotherapy and radiation therapy. A new option for patients with operable invasive breast cancer. Results of a 20 years single institution study. *Ejso*. 2011;37:864–70.

121 Morley KI, Milne RL, Giles GG, *et al.* Socio-economic status and survival from breast cancer for young, Australian, urban women. *Aust N Z J Public Health*. 2010;34:200–5.

122 Morris PG, Murphy CG, Mallam D, *et al.* Limited Overall Survival in Patients with Brain Metastases from Triple Negative Breast Cancer. *Breast Journal*. 2012;18:345–50.

123 Mousavi SA, Kasaeian A, Pourkasmaee M, *et al.* Assessing the prognostic factors, survival, and recurrence incidence of triple negative breast cancer patients, a single center study in Iran. *PLoS One*. 2019;14:e0208701-.

124 Mu L, Liu Y, Xiao M, *et al.* No association between triple-negative breast cancer and prognosis of patients receiving breast-conserving treatment. *Oncol Lett*. 2017;14:7862–72.

125 Mudduwa LK, Wijayaratne GB, Peiris HH, *et al.* Elevated pre-surgical CA15-3: does it predict the short-term disease-free survival of breast cancer patients without distant metastasis? *Int J Womens Health*. 2018;10:329–35.

126 Musolino A, Ciccolallo L, Panebianco M, *et al.* Multifactorial central nervous system recurrence susceptibility in patients with HER2-positive breast cancer: epidemiological and clinical data from a population-based cancer registry study. *Cancer*. 2011;117:1837–46.

127 Nagar H, Boothe D, Ginter PS, *et al.* Disease-free survival according to the use of postmastectomy radiation therapy after neoadjuvant chemotherapy. *Clin Breast Cancer*. 2015;15:128–34.

128 Naher S, Tognela A, Moylan E, *et al.* Patterns of care and outcomes among triple-negative early breast cancer patients in South Western Sydney. *Intern Med J*. 2018;48:567-+.

129 Nedumpara T, Jonker L, Williams MR. Impact of immediate breast reconstruction on breast cancer recurrence and survival. *Breast*. 2011;20:437–43.

130 Nogi H, Kamio M, Toriumi Y, *et al.* Strong impact of pathological node-negative on long-term overall survival of patients with triple-negative breast cancer receiving neoadjuvant chemotherapy. *Mol Clin Oncol*. 2021;14:99.

131 Orditura M, Galizia G, Diana A, *et al.* Neutrophil to lymphocyte ratio (NLR) for prediction of distant metastasis-free survival (DMFS) in early breast cancer: a propensity score-matched analysis. *ESMO Open*. 2016;1.

132 Orsaria P, Grasso A, Ippolito E, *et al.* Clinical outcomes among major breast cancer subtypes after neoadjuvant chemotherapy: Impact on breast cancer recurrence and survival. *Anticancer Res*. 2021;41:2697–709.

133 Park HS, Park S, Kim JH, *et al.* Clinicopathologic Features and Outcomes of Metaplastic Breast Carcinoma: Comparison with Invasive Ductal Carcinoma of the Breast. *Yonsei Med J*. 2010;51:864–9.

134 Park VY, Kim EK, Kim MJ, *et al.* Perfusion Parameters on Breast Dynamic Contrast-Enhanced MRI Are Associated With Disease-Specific Survival in Patients With Triple-Negative Breast Cancer. *American Journal of Roentgenology*. 2017;208:687–94.

135 Peng R, Wang S, Shi Y, *et al.* Patients 35 years old or younger with operable breast cancer are more at risk for relapse and survival: a retrospective matched case-control study. *Breast*. 2011;20:568–73.

136 Perez CA, Zumsteg ZS, Gupta G, *et al.* Black race as a prognostic factor in triple-negative breast cancer patients treated with breast-conserving therapy: a large, single-institution retrospective analysis. *Breast Cancer Res Treat*. 2013;139:497–506.

137 Pomponio MK, Burkbauer L, Goldbach M, *et al.* Refining the indications for neoadjuvant chemotherapy for patients with HER2+ breast cancer: A single institution experience. *J Surg Oncol*. 2020;121:447–55.

138 Prajoko YW, Aryandono T. The Effect of P-Glycoprotein (P-gp), Nuclear Factor-Kappa B (Nf-kappab), and Aldehyde Dehydrogenase-1 (ALDH-1) Expression on Metastases, Recurrence and Survival in Advanced Breast Cancer Patients. *Asian Pac J Cancer Prev*. 2019;20:1511–8.

139 Purushotham A, Shamil E, Cariati M, *et al.* Age at diagnosis and distant metastasis in breast cancer--a surprising inverse relationship. *Eur J Cancer*. 2014;50:1697–705.

140 Qiu J, Xue X, Hu C, *et al.* Comparison of Clinicopathological Features and Prognosis in Triple-Negative and Non-Triple Negative Breast Cancer. *J Cancer*. 2016;7:167–73.

141 Quan ML, Paszat LF, Fernandes KA, *et al.* The Effect of Surgery Type on Survival and Recurrence in Very Young Women With Breast Cancer. *J Surg Oncol*. 2017;115:122–30.

142 Ragage F, Debled M, MacGrogan G, *et al.* Is It Useful to Detect Lymphovascular Invasion in Lymph Node-Positive Patients With Primary Operable Breast Cancer? *Cancer*. 2010;116:3093–101.

143 Raghavan RK, Ibrahim S, Jagathnath Krishna KM, *et al.* Does addition of postmastectomy radiotherapy improve outcome of patients with pT1-2, N0 triple negative breast cancer as compared to breast conservation therapy? *J Cancer Res Ther*. 2019;15:1031–4.

144 Rahal S, Boher JM, Extra JM, *et al.* Immunohistochemical subtypes predict the clinical outcome in high-risk node-negative breast cancer patients treated with adjuvant FEC regimen: results of a single-center retrospective study. *BMC Cancer*. 2015;15.

145 Rakha EA, Martin S, Lee AH, *et al.* The prognostic significance of lymphovascular invasion in invasive breast carcinoma. *Cancer*. 2012;118:3670–80.

146 Raouf SMA, Ibrahim DA, Abdelaziz LA. Immunoexpression of Nanog and Nestin in Egyptian Women Predicts Outcome in Breast Carcinoma. *Journal of Clinical and Diagnostic Research*. 2020;14:EC18–24.

147 Ratosa I, Plavc G, Pislar N, *et al.* Improved Survival after Breast-Conserving Therapy Compared with Mastectomy in Stage I-IIA Breast Cancer. *Cancers (Basel)*. 2021;13.

148 Rosendahl AH, Perks CM, Zeng L, *et al.* Caffeine and Caffeic Acid Inhibit Growth and Modify Estrogen Receptor and Insulin-like Growth Factor I Receptor Levels in Human Breast Cancer. *Clin Cancer Res*. 2015;21:1877–87.

149 Rossi L, Stevens D, Pierga JY, *et al.* Impact of Adjuvant Chemotherapy on Breast Cancer Survival: A Real-World Population. *PLoS One*. 2015;10.

150 Ryu YJ, Kang SJ, Cho JS, *et al.* Lymphovascular invasion can be better than pathologic complete response to predict prognosis in breast cancer treated with neoadjuvant chemotherapy. *Medicine*. 2018;97.

151 Sabiani L, Houvenaeghel G, Heinemann M, *et al.* Breast cancer in young women: Pathologic features and molecular phenotype. *Breast*. 2016;29:109–16.

152 Saha A, Harowicz MR, Cain EH, *et al.* Intra-tumor molecular heterogeneity in breast cancer: definitions of measures and association with distant recurrence-free survival. *Breast Cancer Res Treat*. 2018;172:123–32.

153 Saini A, Kuske R, Quiet C, *et al.* Outcomes by molecular subtype after accelerated partial breast irradiation using single-entry catheters. *Brachytherapy*. 2018;17:415–24.

154 Salem MAE, Hamza HA, El-Aziz N. Neoadjuvant Chemotherapy and Surgical Options for Locally-advanced Breast Cancer: A Single Institution Experience. *Middle East J Cancer*. 2017;8:127–34.

155 Sanchez BC, Sundqvist M, Fohlin H, *et al.* Prolonged tamoxifen treatment increases relapse-free survival for patients with primary breast cancer expressing high levels of VEGF. *Eur J Cancer*. 2010;46:1580–7.

156 Sanpaolo P, Barbieri V, Genovesi D. Prognostic value of breast cancer subtypes on breast cancer specific survival, distant metastases and local relapse rates in conservatively managed early stage breast cancer: A retrospective clinical study. *Ejso*. 2011;37:876–82.

157 Sanpaolo P, Barbieri V, Pedicini P, *et al.* Her-2 prognostic value in very early-stage breast cancer: a single-institution retrospective analysis. *Medical Oncology*. 2012;29:459–65.

158 Sato K, Fuchikami H, Takeda N, *et al.* Efficacy of Single-Plane Implant Technique in Partial Breast Brachytherapy in Small-Breasted Patients. *Int J Radiat Oncol Biol Phys*. 2020;106:830–7.

159 Saw S, Lim J, Lim SH, *et al.* Patterns of relapse after neoadjuvant chemotherapy in breast cancer: implications for surveillance in clinical practice. *Breast Cancer Res Treat*. 2019;177:197–206.

160 Schaffar R, Bouchardy C, Chappuis PO, *et al.* A population-based cohort of young women diagnosed with breast cancer in Geneva, Switzerland. *PLoS One*. 2019;14:e0222136-.

161 Schmidt M, Petry IB, Bohm D, *et al.* Ep-CAM RNA expression predicts metastasis-free survival in three cohorts of untreated node-negative breast cancer. *Breast Cancer Res Treat*. 2011;125:637–46.

162 Schmidt G, Gerlinger C, Juhasz-Boss I, *et al.* Her2-neu score as a prognostic factor for outcome in patients with triple-negative breast cancer. *J Cancer Res Clin Oncol*. 2016;142:1369–76.

163 Shandiz FH, Shabahang H, Afzaljavan F, *et al.* Ki67 Frequency in Breast Cancers without Axillary Lymph Node Involvement and its Relation with Disease-free Survival. *Asian Pac J Cancer Prev*. 2016;17:1347–50.

164 Sharma P, Lopez-Tarruella S, Garcia-Saenz JA, *et al.* Pathological Response and Survival in Triple-Negative Breast Cancer Following Neoadjuvant Carboplatin plus Docetaxel. *Clin Cancer Res*. 2018;24:5820–9.

165 Sharp NE, Sachs DB, Melchior NM, *et al.* Does the false-negative rate for 1 or 2 negative sentinel nodes after neo-adjuvant chemotherapy translate into a high local recurrence rate? *Breast J*. 2021;27:335–44.

166 Shen HH, Yuan JY, Yuan S, *et al.* Survival estimates based on molecular subtype and age in patients with early node-negative breast cancer. *Int J Clin Exp Pathol*. 2016;9:5357–67.

167 Shiao J, Thomas KM, Rahimi AS, *et al.* Aspirin/antiplatelet agent use improves disease-free survival and reduces the risk of distant metastases in Stage II and III triple-negative breast cancer patients. *Breast Cancer Res Treat*. 2017;161:463–71.

168 Shim B, Jin MS, Moon JH, *et al.* High Cytoplasmic CXCR4 Expression Predicts Prolonged Survival in Triple-Negative Breast Cancer Patients Treated with Adjuvant Chemotherapy. *J Pathol Transl Med*. 2018;52:369–77.

169 Shohdy KS, Almeldin DS, Fekry MA, *et al.* Pathological responses and survival outcomes in patients with locally advanced breast cancer after neoadjuvant chemotherapy: a single-institute experience. *J Egypt Natl Canc Inst*. 2021;33:39.

170 Simons JM, Jacobs JG, Roijers JP, *et al.* Disease-free and overall survival after neoadjuvant chemotherapy in breast cancer: breast-conserving surgery compared to mastectomy in a large single-centre cohort study. *Breast Cancer Res Treat*. 2021;185:441–51.

171 Smith BL, Tang R, Rai U, *et al.* Oncologic Safety of Nipple-Sparing Mastectomy in Women with Breast Cancer. *J Am Coll Surg*. 2017;225:361–5.

172 Song YJ, Shin SH, Cho JS, *et al.* The Role of Lymphovascular Invasion as a Prognostic Factor in Patients with Lymph Node-Positive Operable Invasive Breast Cancer. *J Breast Cancer*. 2011;14:198–203.

173 Sopik V, Sun P, Narod SA. Predictors of time to death after distant recurrence in breast cancer patients. *Breast Cancer Res Treat*. 2019;173:465–74.

174 Soran A, Ozmen T, Salamat A, *et al.* Lymph Node Ratio (LNR): Predicting Prognosis after Neoadjuvant Chemotherapy (NAC) in Breast Cancer Patients. *Eur J Breast Health*. 2019;15:249–55.

175 Spring L, Greenup R, Niemierko A, *et al.* Pathologic Complete Response After Neoadjuvant Chemotherapy and Long-Term Outcomes Among Young Women With Breast Cancer. *Journal of the National Comprehensive Cancer Network*. 2017;15:1216–23.

176 Sun J, Mathias BJ, Laronga C, *et al.* Impact of Axillary Dissection Among Patients With Sentinel Node-Positive Breast Cancer Undergoing Mastectomy. *J Natl Compr Canc Netw*. 2021;19:40–7.

177 Tjokrowidjaja A, Lee CK, Houssami N, *et al.* Metastatic breast cancer in young women: a population-based cohort study to describe risk and prognosis. *Intern Med J*. 2014;44:764–70.

178 Tokuda E, Horimoto Y, Arakawa A, *et al.* Differences in Ki67 expressions between pre- and post-neoadjuvant chemotherapy specimens might predict early recurrence of breast cancer. *Hum Pathol*. 2017;63:40–5.

179 Tong Y, Wu J, Huang O, *et al.* IGF-1 Interacted With Obesity in Prognosis Prediction in HER2-Positive Breast Cancer Patients. *Front Oncol*. 2020;10:550.

180 Tonyali O, Coskun U, Sener N, *et al.* Prognostic factors for recurrence-free survival in patients with HER2-positive early-stage breast cancer treated with adjuvant trastuzumab. *Onkologie*. 2013;36:554–8.

181 Tovanabutra C, Katanyoo K, Uber P, *et al.* Comparison of Treatment Outcome between Hypofractionated Radiotherapy and Conventional Radiotherapy in Postmastectomy Breast Cancer. *Asian Pac J Cancer Prev*. 2020;21:119–25.

182 Tung N, Gaughan E, Hacker MR, *et al.* Outcome of triple negative breast cancer: comparison of sporadic and BRCA1-associated cancers. *Breast Cancer Res Treat*. 2014;146:175–82.

183 Tzikas AK, Nemes S, Linderholm BK. A comparison between young and old patients with triple-negative breast cancer: biology, survival and metastatic patterns. *Breast Cancer Res Treat*. 2020;182:643–54.

184 Ulas A, Kos T, Avci N, *et al.* Patients with HER2-positive early breast cancer receiving adjuvant trastuzumab: clinicopathological features, efficacy, and factors affecting survival. *Asian Pac J Cancer Prev*. 2015;16:1643–9.

185 Van Asten K, Slembrouck L, Olbrecht S, *et al.* Prognostic Value of the Progesterone Receptor by Subtype in Patients with Estrogen Receptor-Positive, HER-2 Negative Breast Cancer. *Oncologist*. 2019;24:165–71.

186 Vu Hong T, Nguyen Ba D, Skoog L, *et al.* Breast Cancer Survival Defined by Biological Receptor and Menopausal Status in Vietnamese Women. *Cancer Control*. 2019;26:1073274819865279-.

187 Wang J, Li Q, Zhang P, *et al.* Young breast cancer patients who develop distant metastasis after surgery have better survival outcomes compared with elderly counterparts. *Oncotarget*. 2017;8:44851–9.

188 Wang X, Yin Z, Wang D, *et al.* Greater negative lymph node count predicts favorable survival of patients with breast cancer in the setting of neoadjuvant chemotherapy and mastectomy. *Future Oncol*. 2019;15:3701–9.

189 Wang SL, Wen G, Tang Y, *et al.* Effectiveness of the AJCC 8th edition staging system for selecting patients with T1-2N1 breast cancer for post-mastectomy radiotherapy: a joint analysis of 1986 patients from two institutions. *BMC Cancer*. 2020;20.

190 Wang L, Zhang Y, He Y, *et al.* Impact of dose-dense neoadjuvant chemotherapy on pathologic response and survival for HER2-positive breast cancer patients who receive trastuzumab. *NPJ Breast Cancer*. 2021;7:75.

191 Wen HY, Krystel-Whittemore M, Patil S, *et al.* Breast carcinoma with an Oncotype Dx recurrence score <18: Rate of distant metastases in a large series with clinical follow-up. *Cancer*. 2017;123:131–7.

192 White R, Dinneen T, Makris A. Local radiotherapy alone following neoadjuvant chemotherapy and surgery in combined clinical stage II and III breast cancer. *Radiation Oncology*. 2016;11.

193 Wirtz HS, Buist DS, Gralow JR, *et al.* Frequent antibiotic use and second breast cancer events. *Cancer Epidemiol Biomarkers Prev*. 2013;22:1588–99.

194 Wobb JL, Shah C, Chen PY, *et al.* Brachytherapy-based Accelerated Partial Breast Irradiation Provides Equivalent 10-Year Outcomes to Whole Breast Irradiation: A Matched-Pair Analysis. *Am J Clin Oncol*. 2016;39:468–72.

195 Wu S, Zhou J, Ren Y, *et al.* Tumor location is a prognostic factor for survival of Chinese women with T1-2N0M0 breast cancer. *Int J Surg*. 2014;12:394–8.

196 Wu S, Li Q, Zhou J, *et al.* Post-mastectomy radiotherapy can improve survival in breast cancer patients aged 35 years or younger with four or more positive nodes but not in one to three positive nodes. *Ther Clin Risk Manag*. 2014;10:867–74.

197 Wu X, Baig A, Kasymjanova G, *et al.* Pattern of Local Recurrence and Distant Metastasis in Breast Cancer By Molecular Subtype. *Cureus*. 2016;8:e924-.

198 Xu FF, Cao L, Xu C, *et al.* Practical Model to Optimize the Strategy of Adjuvant Postmastectomy Radiotherapy in T1-2N1 Breast Cancer With Modern Systemic Therapy. *Front Oncol*. 2022;12:789198.

199 Xue C, Peng RJ, Wang SS, *et al.* Operable breast cancer of the inner hemisphere is associated with poor survival. *J Breast Cancer*. 2015;18:36–43.

200 Yamada Y, Mukai H, Tokudome Y, *et al.* Improved overall survival over recent decades in patients with hormone-receptor-positive, HER2-negative breast cancer: a single-center retrospective analysis of prognostic factors. *Jpn J Clin Oncol*. 2018;48:248–54.

201 Yamada A, Narui K, Satake T, *et al.* Long-Term Outcomes of Immediate Autologous Breast Reconstruction for Breast Cancer Patients. *J Surg Res*. 2020;251:78–84.

202 Yang J, Tang S, Zhou Y, *et al.* Prognostic implication of the primary tumor location in early-stage breast cancer: focus on lower inner zone. *Breast Cancer*. 2018;25:100–7.

203 Ye F, Huang L, Lang G, *et al.* Outcomes and risk of subsequent breast events in breast-conserving surgery patients with BRCA1 and BRCA2 mutation. *Cancer Med*. 2020;9:1903–10.

204 Yoon CI, Ahn SG, Bae SJ, *et al.* High A20 expression negatively impacts survival in patients with breast cancer. *PLoS One*. 2019;14.

205 Zaky SS, Lund M, May KA, *et al.* The Negative Effect of Triple-Negative Breast Cancer on Outcome after Breast-Conserving Therapy. *Ann Surg Oncol*. 2011;18:2858–65.

206 Zemni I, Ghalleb M, Jbir I, *et al.* Identifying accessible prognostic factors for breast cancer relapse: a case-study on 405 histologically confirmed node-negative patients. *World J Surg Oncol*. 2017;15.

207 Zhang Y, Chen Y, Chen D, *et al.* Impact of preoperative anemia on relapse and survival in breast cancer patients. *BMC Cancer*. 2014;14:844.

208 Zhang WW, Wu SG, Sun JY, *et al.* Long-term survival effect of the interval between mastectomy and radiotherapy in locally advanced breast cancer. *Cancer Manag Res*. 2018;10:2047–54.

209 Zhang Y, Zhang Y, Liu Z, *et al.* Impact of Postmastectomy Radiotherapy on Locoregional Control and Disease-Free Survival in Patients with Breast Cancer Treated with Neoadjuvant Chemotherapy. *J Oncol*. 2021;2021:6632635.

210 Zhao Y, Dong X, Li R, *et al.* Correlation Between Clinical-Pathologic Factors and Long-Term Follow-Up in Young Breast Cancer Patients. *Transl Oncol*. 2015;8:265–72.

211 Zhong W, Tan L, Jiang WG, *et al.* Effect of younger age on survival outcomes in T1N0M0 breast cancer: A propensity score matching analysis. *J Surg Oncol*. 2019;119:1039–46.

212 Zouzoulas D, Tsolakidis D, Gitas G, *et al.* Breast cancer in women younger than 35 years old. *Arch Gynecol Obstet*. 2020;302:721–30.

213 Zumsteg ZS, Morrow M, Arnold B, *et al.* Breast-conserving therapy achieves locoregional outcomes comparable to mastectomy in women with T1-2N0 triple-negative breast cancer. *Ann Surg Oncol*. 2013;20:3469–76.

214 van Steenhoven JEC, Kuijer A, van Maaren MC, *et al.* Quantifying the Mitigating Effects of Whole-Breast Radiotherapy and Systemic Treatments on Regional Recurrence Incidence Among Breast Cancer Patients. *Ann Surg Oncol*.

215 van Laar C, van der Sangen MJ, Poortmans PM, *et al.* Local recurrence following breast-conserving treatment in women aged 40 years or younger: trends in risk and the impact on prognosis in a population-based cohort of 1143 patients. *Eur J Cancer*. 2013;49:3093–101.

216 van Roozendaal LM, Smit LHM, Duijsens G, *et al.* Risk of regional recurrence in triple-negative breast cancer patients: a Dutch cohort study. *Breast Cancer Res Treat*. 2016;156:465–72.

217 van den Hurk CJ, Eckel R, van de Poll-Franse L V, *et al.* Unfavourable pattern of metastases in M0 breast cancer patients during 1978-2008: a population-based analysis of the Munich Cancer Registry. *Breast Cancer Res Treat*. 2011;128:795–805.
